# Assessing eco-geographic influences on COVID-19 transmission: a global analysis

**DOI:** 10.1038/s41598-024-62300-y

**Published:** 2024-05-22

**Authors:** Jing Pan, Arivizhivendhan Kannan Villalan, Guanying Ni, Renna Wu, ShiFeng Sui, Xiaodong Wu, XiaoLong Wang

**Affiliations:** 1 Key Laboratory for Wildlife Diseases and Bio-Security Management of Heilongjiang Province, Heilongjiang Province, Harbin, 150040 People’s Republic of China; 2 College of Wildlife and Protected Area, Northeast Forestry University, Heilongjiang Province, Harbin, 150040 People’s Republic of China; 3 HaiXi Animal Disease Control Center, Qinghai Province, Delingha, 817099 People’s Republic of China; 4 Zhaoyuan Forest Resources Monitoring and Protection Service Center, Shandong Province, Zhaoyuan, 265400 People’s Republic of China; 5 China Animal Health and Epidemiology Center, Shandong Province, Qingdao, 266032 People’s Republic of China

**Keywords:** COVID-19, Epidemiological characteristics analysis, Spatial modeling, Maximum entropy, Population-based studies, Risk assessment, Ecology, Ecology, Environmental social sciences

## Abstract

COVID-19 has been massively transmitted for almost 3 years, and its multiple variants have caused serious health problems and an economic crisis. Our goal was to identify the influencing factors that reduce the threshold of disease transmission and to analyze the epidemiological patterns of COVID-19. This study served as an early assessment of the epidemiological characteristics of COVID-19 using the MaxEnt species distribution algorithm using the maximum entropy model. The transmission of COVID-19 was evaluated based on human factors and environmental variables, including climate, terrain and vegetation, along with COVID-19 daily confirmed case location data. The results of the SDM model indicate that population density was the major factor influencing the spread of COVID-19. Altitude, land cover and climatic factor showed low impact. We identified a set of practical, high-resolution, multi-factor-based maximum entropy ecological niche risk prediction systems to assess the transmission risk of the COVID-19 epidemic globally. This study provided a comprehensive analysis of various factors influencing the transmission of COVID-19, incorporating both human and environmental variables. These findings emphasize the role of different types of influencing variables in disease transmission, which could have implications for global health regulations and preparedness strategies for future outbreaks.

## Introduction

COVID-19 (Corona Virus Disease 2019) has become a global public health threat. Coronavirus Disease 2019 (COVID-19) is a severe acute respiratory syndrome caused by coronavirus type 2 (SARS-CoV-2), which emerged in December 2019. The World Health Organization declared it a global pandemic on March 11, 2020^1,2^. Coronaviruses are a large group of viruses, some of which can cause respiratory diseases in humans and often trigger serious global public health crises. The coronavirus (CoV) belongs to the family Coronaviridae and is a single-stranded envelope virus with an RNA genome size of approximately 26–32 kb^3^. SARS-CoV-2 can be transmitted from person to person through droplets, aerosols, and contact^4^. Common clinical manifestations include fever, cough, fatigue, difficulty breathing, diarrhea, nausea, and vomiting^4–6^. Moreover, SARS-CoV-2 infection may lead to long-term lung damage and relatively frequent cardiac involvement^7^. The SARS-CoV-2 quickly spread worldwide within a few months, leading to global panic and conflicts of interest^8,9^. As of March 1, 2023, the global confirmed cases of COVID-19 were 676 million, with 6.87 million deaths in 188 countries/regions^10^. Research suggests that the actual number of deaths is higher, which was estimated to be as high as 20 million as of 2022^11,12^. Although the new generation vaccines and anti-COVID-19 treatment schemes prove helpful in managing acute COVID-19 infection, scientists express concern that the persistent unvaccinated population globally may pose a greater risk for the emergence of new mutated strains, such as Omicron^13^. Highly transmissible variants to a certain extent hinder the suppression of the vaccine against the spread of COVID-19^14^, monitoring of mutated strains remains largely inadequate, with an incomplete understanding of the risk of reinfection^15,16^. Many public health experts still believe that COVID-19 is an ongoing health threat^17^. COVID-19 has become a serious chronic disease globally at present and even in the next few years that constitutes a considerable disease burden^18,19^, but it still lacks sufficient awareness. Understanding the influence of each factor on the transmission of catastrophic threats like COVID-19 is crucial for successful policy implementation and risk management to control the outbreak^20^.

The impact of environmental and human factors on the transmission of COVID-19 has been a significant question since the beginning of the pandemic^20–22^. Various meteorological factors, such as temperature and humidity, influenced the infection rate of respiratory viruses and host immunity, leading to variations in the spread of respiratory viruses in different regions^22^. Sajadi et al. conducted research on 50 cities throughout the world and found that cities with widespread community transmission were mostly distributed between 30°N and 50°N with temperatures ranging from 5 to 11 °C^23^. The epidemic transmission trajectory of many countries shows strong seasonal patterns, with fewer cases in summer and more cases in winter^24^. The temperature increase from 22 to 34 °C significantly activated the virus-like particles (VLPs), causing damage to the stability of the virus^25^. Higher transmissibility is likely to be seen at low temperatures, while higher severity is likely to present at high and moderately low temperatures in Japan^26^, while there was no significant correlation between temperature and COVID-19 in Spain, which gives an opposite conclusion^27^. Similarly, humidity has a negative correlation or no correlation with COVID-19 cases. A study by Wu et al. demonstrated a negative correlation between COVID-19 cases and humidity levels^28^, that high temperature and high relative humidity reduce the viability, stability, survival, and transmission of COVID-19^29^. A study found that there is no correlation between humidity and COVID-19 cases in Pakistan^30^. A large number of studies have explored the relationship between the spread of COVID-19 and meteorological factors, but it is still controversial^31^. Areas with lower solar radiation showed high exposure rate^32^. Solar radiation can destroy the genetic material of viruses, such as DNA or RNA, thus threatening the survival of viruses^33^. The daily growth rates of cumulative COVID-19 deaths decreased by 1.2% with each unit increase in the UV index^34^.

The influence of meteorological drivers on COVID-19 transmission globally is confounded by other factors, such as altitude, population density, and land cover. Previous researches have revealed that population density was more important than meteorological factors^35^. High population density is more likely to lead to the outbreak of severe acute respiratory COVID-19^36^. Because the respiratory virus is mainly transmitted through the respiratory tract, the higher the population density, the longer the time for the spread and attenuation of COVID-19^37^. The research of Nasiri in Iran indicates that the number of patients is higher in areas with high population density and commercial and residential land^38^. The natural environment is positively correlated with public health^39,40^. More green spaces in the short term are also associated with lower morbidity and mortality rates^41^. The study underscores the importance of incorporating natural land cover as a means of mitigating the risks and negative consequences of future pandemics like COVID-19 and promoting overall public health. Meanwhile, available epidemiological data suggest a negative correlation between altitude and the incidence of COVID-19^42^. The city size and population density of high-altitude regions are lower than in low-altitude regions, which decreases the mobility of high-altitude regions, thereby reducing the transmission of the pandemic in high-altitude regions^43^.

So far, despite numerous studies on the impact of various factors on the spread of COVID-19, few studies have simultaneously considered meteorological variables, population factors, land cover types, altitude, and other terrain factors to investigate their combined influence on the development of COVID-19. In addition, most studies are limited to a single country or region, but the understanding of the relationship between them is relatively limited on a global scale. Moreover, most of the research data worldwide is based on national sources, which are not accurate enough. Therefore, a comprehensive assessment is needed to understand the dynamics of COVID-19 transmission worldwide. Systematic and scientific research on the epidemic characteristics, influencing factors, and transmission risks of newly emerging infectious diseases, could better establish an early warning system, predict the future pandemic trends, and avoid more public health losses.

Risk prediction is an important measure for controlling and preventing outbreaks of infectious diseases, and has been used to draw COVID-19 epidemic maps, which are methods to deeply reveal the dynamics of the virus and have powerful functions in establishing disease transmission models, detecting important hotspots, and predicting the occurrence of diseases in the future. Among numerous niche models, MaxEnt has been widely used due to its advantages^44,45^. It has been widely used in many diseases, including COVID-19^3,46,47^. To overcome the above shortcomings, the purpose of this study is to conduct a comprehensive analysis based on geographically narrow data sources, use the MaxEnt model to assess the collective impact of the above factors on COVID-19 cases, and further explore the differences in these impacts in different regions. We gathered global COVID-19 case data at a city scale along with population density, land cover, altitude, solar radiation, and climate factors. MaxEnt, ArcGIS, and SPSS were used to deeply explore the impact of meteorological, population density, and other relevant factors on the spread of COVID-19. The main objective of the study is to explore the potential interaction and identification of COVID-19 risk areas and hotspots at a global scale, in order to provide guidance for the scientific prevention and control of the COVID-19 outbreak. This research would provide useful guidance for local health authorities in deciding where to prioritize effective interventions on a fine scale.

## Results

**Figure 1:**
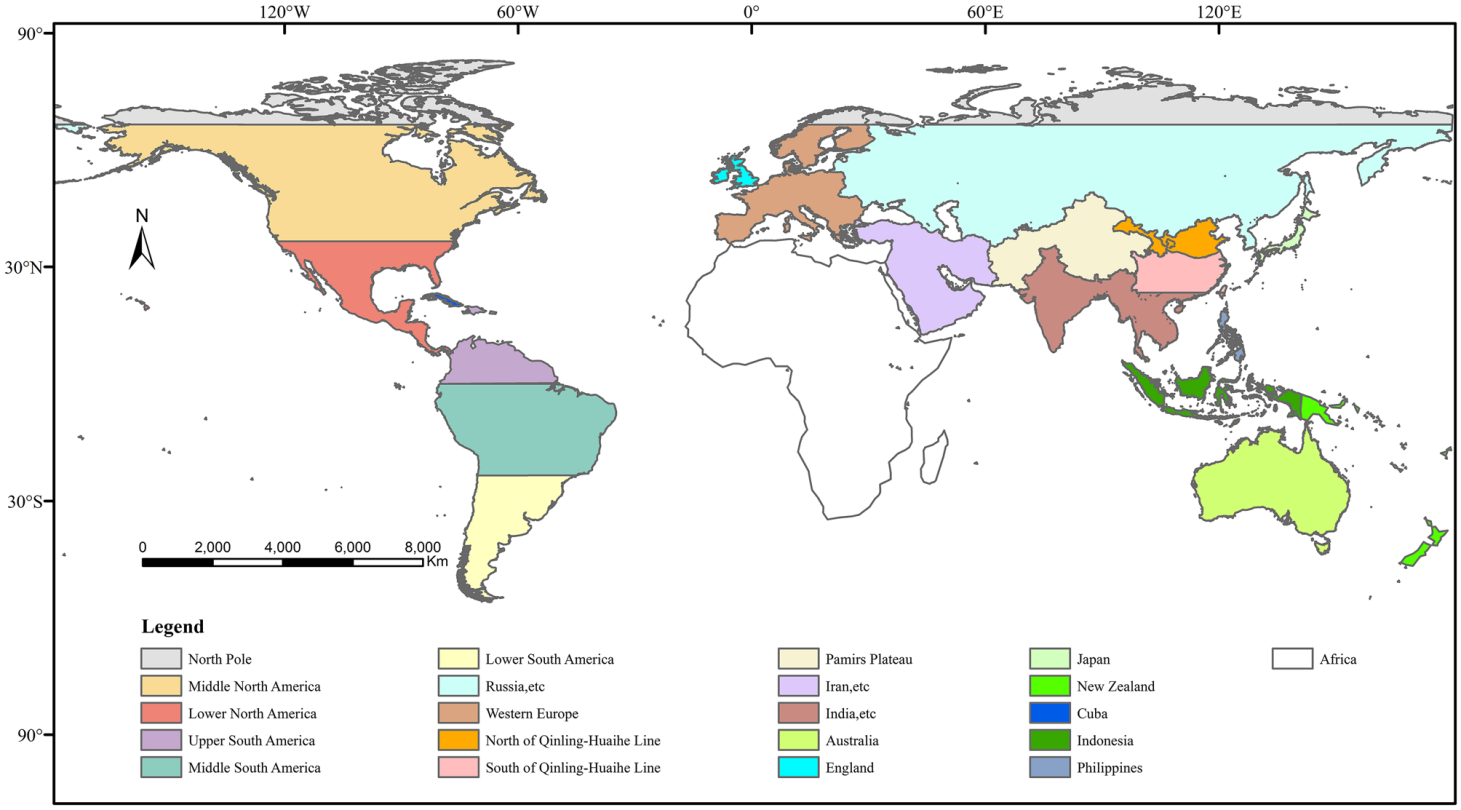
Global map classification based on geographical regions sourced from Natural Earth (http://www.naturalearthdata.com/).

An early assessment of the epidemiological characteristics of SARS-CoV-2 was conducted using the MaxEnt species distribution algorithm to study the future risk distribution of COVID-19 infection risk hotspots. A global map was classified based on geographical regions sourced from Natural Earth (http://www.naturalearthdata.com/) and used for the MaxEnt model (Fig. 1). A total of 28,142 COVID-19 occurrence points were selected after filtering for application in the MaxEnt to evaluate the future possible risk distribution of COVID-19. The model parameters were optimized and evaluated for the effective prediction of COVID-19 distribution. The ROC curves of prediction results from the MaxEnt models regarding sensitivity and specificity are in Figures S19–S22. The average result obtained from the tenfold cross‐validation of the COVID-19 species distribution model (SDM) revealed that the average AUC value of 28 models was above 0.8, wherein, 19 models were above 0.9.

### Rarefying and variables selection

**Figure 2:**
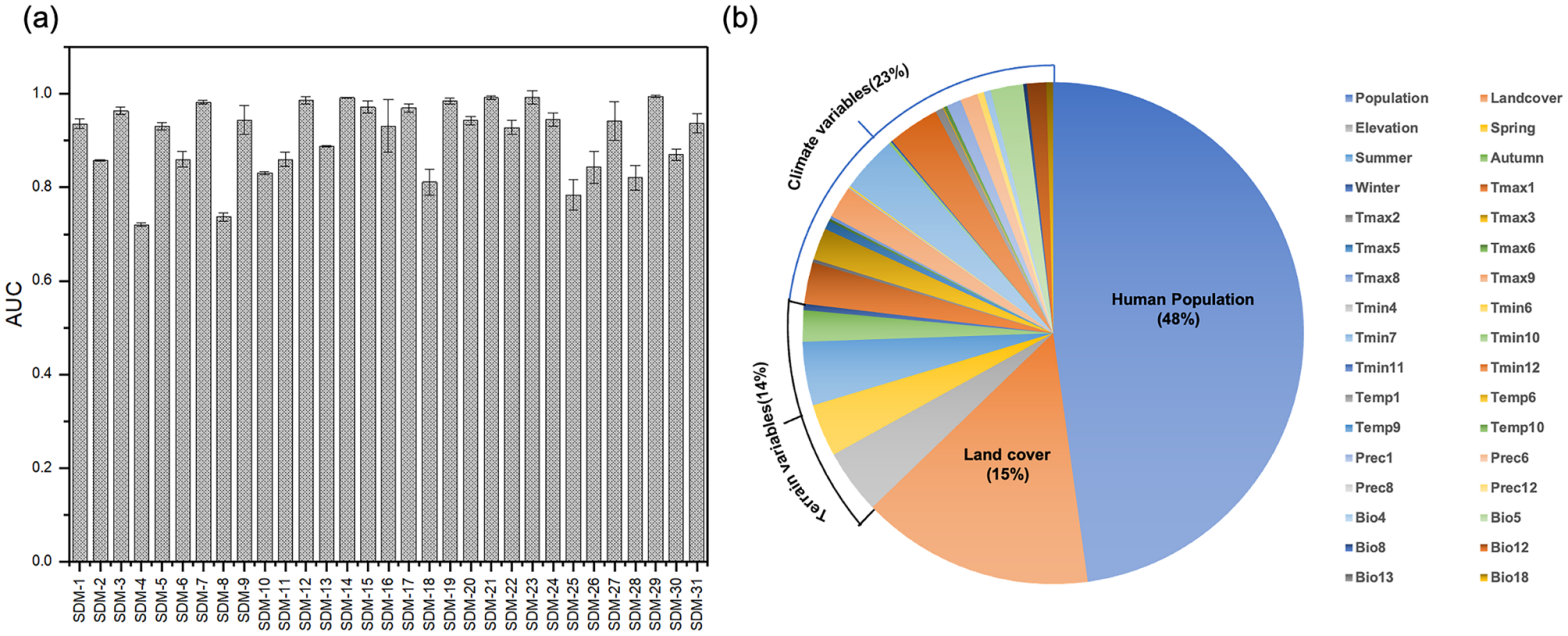
(**a**) AUC values of MaxEnt models and (**b**) contributions of important predictor variables to the mod.

The accuracy of SDM model was validated based on AUC values, with the expectation that the best model would have an AUC value near 1. The average output result of the tenfold cross‐validation of the COVID-19 in SDM model demonstrated high training and test AUC values, combined with low standard deviations. The results indicated that the average AUC value for all research areas ranged from 0.711 to 0.994 (Fig. 2a). Among the 31 models, only three models, such as SDM4, SDM8, and SDM28, had AUC values below 0.8 although they still exceeded 0.7. This suggests that the accuracy of the models was ‘very good’, and the prediction results were reliable, enabling the prediction of COVID-19 distribution. The results of the MaxEnt software simulation output ranged from 0 to 1, where values were closer to 1 corresponded to a higher probability of species existence. The environmental variables and mean range of VIF value for all niche models were provided in Table 1. The natural break was used as the minimum distance allowed between training points for the spatially filtered occurrence dataset for spotted knapweed. The application of this minimum distance in spatial filtering led to significant reduction in training sample size (Table 1).

**Table 1:** The VIF value of all models and its detailed information of geographical region, COVID-19 occurrence point, elevation and environment variables.

Models	Regions	COVID-19 presence points	Elevation	Natural break	Environmental layers	VIF
SDM-1	North pole	105	Below 1500	10 km	Population, land cover and maximum temperature of March (tmax3)	1.014–1.742
SDM-2	Middle North America	1980	Below 1500	10 km	Population and maximum temperature of May (tmax5)	1.213–3.642
SDM-3	Middle North America	1276	Above 1500	10 km	Minimum temperature of July (tmin7), spring, population and land cover	1.009–2.931
SDM-4	Lower North America	2170	Below 1500	10 km	Population, elevation, land cover, minimum temperature of November (tmin11) and mean temperature of March (temp3)	1.038–9.558
SDM-5	Lower North America	823	Above 1500	10 km	Minimum temperature of July (tmin7), spring, population and land cover	1.000
SDM-6	Upper Sorth America	879	Below 1500	10 km	Population, land cover and autumn	1.000–3.409
SDM-7	Upper South America	11	Above 1500	10 km	Population, autumn, mean temperature of October (temp10) and land cover	1.0001–5.584
SDM-8	Middle Sorth America	3940	Below 1500	10 km	Land cover, population, mean temperature of June (temp6) and autum	1.000
SDM-9	Middle South America	11	Above 1500	10 km	Mean temperature of October (temp10), land cover and elevation	0.965–0.993
SDM-10	Lower Sorth America	1753	Below 1500	10 km	Population, land cover and maximum temperature of January (tmax1)	1.000
SDM-11	Western Europe	105	Below 1500	160 km	Elevation, population, land cover, winter, maximum temperature of August (tmax8) and Precipitation of Wettest Month (bio13)	1.014–1.078
SDM-12	Western Europe	41	Above 1500	10 km	Minimum temperature of July (tmin7), precipitation of June (prec6), winter, land cover and population	1.000
SDM-13	Russia,etc	1224	Below 1500	40 km	Population the mean monthly Precipitation of Warmest Quarter (bio18) and summer	1.000
SDM-14	Russia,etc	28	Above 1500	20 km	The mean monthly precipitation amount of the wettest quarter (bio16), population and land cove	1.001–6.921
SDM-15	Iran,etc.;	103	Above 1500	10 km	Population, summer, the Mean Temperature of Wettest Quarter (bio8), and minimum temperature of December (tmin12)	1.001–1.233
SDM-16	Pamirs Plateau	32	Below 1500	50 km	Population, land cover, precipitation of December (prec12) and minimum temperature of Octoberber (tmin10)	1.000–1.005
SDM-17	Pamirs Plateau	25	Above 1500	10 km	Land cover, population and the precipitation amount of January (prec1)	1.000–1.765
SDM-18	India, etc	85	Below 1500	10 km	The mean monthly precipitation amount of the wettest quarter (bio16), population and land cover	1.000
SDM-19	India, etc	7	Above 1500	10 km	Bio5, Tmax3 maximum temperature of March, Bio4	1.000
SDM-20	Up Qinling-Huaihe Line	367	Below 1500	10 km	Population, land cover, elevation, precipitation of August (prec8) summer	1.000–1.911
SDM-21	Up Qinling-Huaihe Line	18	Above 1500	10 km	Land cover, spring, maximum temperature of January (tmax1), population	1.000
SDM-22	Blow Qinling-Huaihe Line	717	Below 1500	10 km	Population, land cover, elevation	1.000–1.009
SDM-23	Blow Qinling-Huaihe Line	20	Above 1500	10 km	Population, elevation, land cover	1.000
SDM-24	Australia	289	Below 1500	10 km	Population, mean temperature of January (temp1)	1.000
SDM-25	Cuba	47	Below 1500	40 km	Land cover, population, precipitation amount of June (prec6) and minimum temperature of April (tmin4)	1.007–1.794
SDM-26	England	60	Below 1500	50 km	Population, land cover, maximum temperature of June (tmax6), minimum temperature of June (tmin6), summer	1.011–1.116
SDM-27	Japan	58	Below 1500	20 km	Population, maximum temperature of January (tmax1), land cover	1.000
SDM-28	The Philippines	69	Below 1500	110 km	Maximum temperature of September (tmax9), land cover, precipitation amount of January (prec1), population	1.136–5.862
SDM-29	The Philippines	8	Above 1500	0 km	Minimum temperature of June (tmin12), land cover	1.000
SDM-30	Indonisia	163	Below 1500	60 km	Population, land cover, maximum temperature of February (tmax2), spring	1.000–2.005
SDM-31	New Zealand	20	Below 1500	10 km	Population, maximum temperature of September (tmax9), land cover, mean temperature of September (temp9)	1.000

### Influence of population density on COVID-19

**Figure 3:**
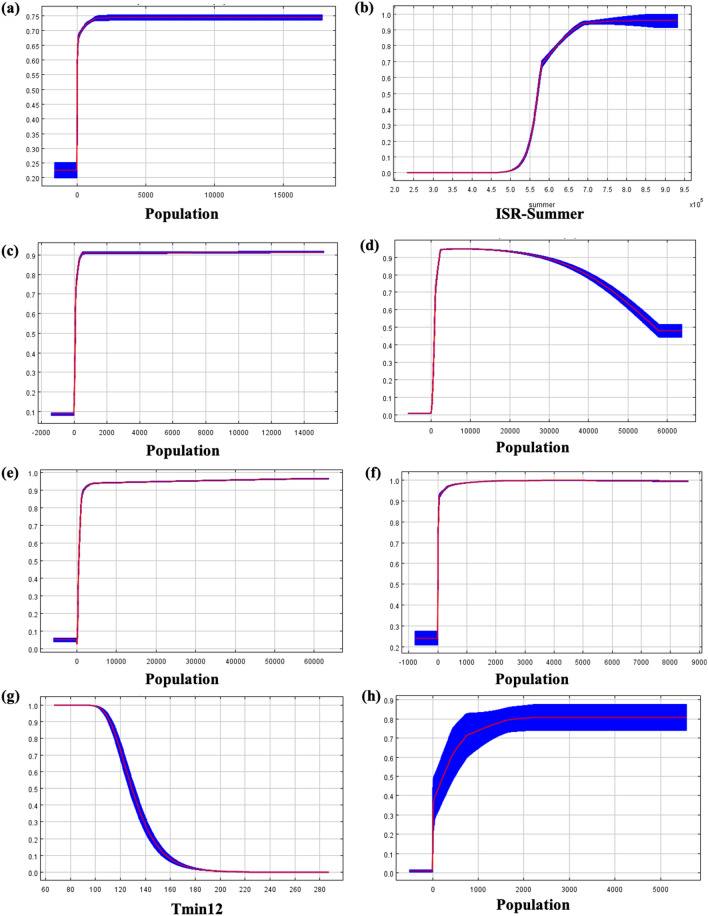
Analysis of COVID-19 distribution models' response curves influenced by factors which contribution rate is more than 80%. The models (**a**) SDM2, (**b**) SDM3, (**c**) SDM6, (**d**) SDM20, (**e**) SDM22, (**f**) SDM24, (**g**) SDM29 and (**h**) SDM (31).

The result revealed that population density variables significantly influenced the transmission of COVID-19 more than other variables (Fig. 2b). The influence of population density on risk distribution areas was notably high in most of the models (Fig. 2b). The SDM-31 had the highest impact at 93.2%, followed by SDM-20 (92.9%), SDM-22 (91%), SDM-6 (88.5%), SDM-24 (84.3%), SDM-2 (82.5%), SDM-30 (77.9%), SDM-13 (77.7%), SDM-15 (70.3%), SDM-9 (65.2%), SDM-23 (62.8%), SDM-1 (62.2%), SDM-10 (60.1%), SDM-7 (55.2%), SDM-16 (54.3%), SDM-26 (54.2%), SDM-4 (45.9%), SDM-27 (39.6%), SDM-8 (37.8%), SDM-14 (36.6%), SDM-25 (34.5%), SDM-11 (29.5%), SDM-17 (22.9%), SDM-5 (19.3%), SDM-18 (15.4%), SDM-21 (11.9%) (Tables 2 and 3). Out of a total of 31 SDM models, 8 SDM models contributed more than 80% to the specified environmental and geographic variables, and 6 of these SDM models were highly influenced by population density (Fig. 3). The population density factor significantly influenced both mainland and island countries in most of the models, except for two niche models. The MaxEnt response curves of each model predictor are shown in Figures S2–S13. The population density in New Zealand significantly impacts the distribution of SARS-CoV-2, with an estimated contribution of up to 93.2% (Table 2). The distribution probability of SARS-CoV-2 becomes stable when the population density reaches 2000 people/km^2^. Similarly, estimates of contribution above 80% were reported for regions in upper South America, Australia, and Middle North America. In most areas below 1500 m of elevation, such as India and Western Europe, an increase in population density led to a significant reduction in the distribution probability of SARS-CoV-2. The distribution probability of COVID-19 increased sharply with the increase in population density in most regions when the elevation was below 1500 m.

**Table 2:** Percentage contributions of predictor variables to the MaxEnt models blow than 1500 m.

	SDM-1	SDM-2	SDM-4	SDM-6	SDM-8	SDM-10	SDM-11	SDM-13	SDM-16	SDM-18	SDM-20	SDM-22	SDM-24	SDM-25	SDM-26	SDM-27	SDM-28	SDM-30	SDM-31
Population	62.2	82.5	45.9	88.5	37.8	60.1	29.5	77.7	54.3	15.4	92.9	91	84.3	34.5	54.2	39.6	6.5	77.9	93.2
Landcover	10.2		8.4	6.6	40.1	23	19.2		27.3	25.7	6	6.3		43.7	38	29	23.4	9.4	2.6
Elevation			42.6				38.8				0.7	2.8							
Spring										5.8				2.3				5.8	
Summer								8.7			0.1				0.3				
Autumn				4.9	19.7														
Winter							3.3												
Tmax1						16.9				17.9						31.4			
Tmax2																		6.9	
Tmax3	27.6																		
Tmax5		17.5																	
Tmax6															4.6				
Tmax8							5.9												
Tmax9																	60.1		3.5
Tmin4														2.9					
Tmin6															2.9				
Tmin10									5.2										
Tmin11			3.2																
Temp1													15.7						
Temp6					2.4														
Temp9																			0.7
Prec1																	10.1		
Prec6														16					
Prec8											0.3								
Prec12									13.3										
Bio12										35.1									
Bio13							3.3												
Bio18								13.6

**Table 3:** Percentage contributions of predictor variables to the MaxEnt models above than 1500 m.

	SDM-3	SDM-5	SDM-7	SDM-9	SDM-12	SDM-14	SDM-15	SDM-17	SDM-19	SDM-21	SDM-23	SDM-29
Population		19.3	55.2		9.1	36.6	70.3	22.9		11.9	62.8	
Landcover		1.7	0.8	25.7	8.8			58		45.7	2.3	3.5
Elevation				9.1							34.9	
Spring		65.8								25.6		
Summer	84.9					17.2	15.7					
Autumn			37.9									
Winter					9.2							
Tmax1										16.7		
Tmax3	15.1								20.9			
Tmin7		13.2			54.2	46.3						
Tmin12							6.7					96.5
Temp10			6	65.2								
Prec1								19.1				
Prec6					18.8							
Bio4									13.8			
Bio5									65.3			
Bio8							7.2

### Influence of land cover and elevation variables on COVID-19

The probability of COVID-19 distribution was not influenced by population density factor in some regions, such as those with elevation greater than 1500 m in the Philippines, Middle North America, and India (Figures S3, S9, and S12). In areas with elevations above 1500 m, the contribution rate of population density was relatively lower (Figure S2b).

The proportions of altitude and land cover showed a significant influence on the probability of COVID-19 distribution (Fig. 2b). In regions below 1500 m altitude, the land cover had a significant impact on these models, followed by the impact of population density (Figure S2a). In regions above 1500 m altitude, the terrain variables showed a significant impact. The land cover relatively influences the probability of COVID-19 distribution models such as SDM-17 (58%), SDM-21 (45.7%), SDM-25 (43.7%), SDM-8 (40.1%), SDM-26 (38%), SDM-27 (29%), SDM-16 (27.3%), SDM-9 (25.7%), SDM-18 (25.7%), SDM-28 (23.4%), SDM-10 (23%), SDM-11 (19.2%) and SDM-1 (10.2%) (Tables 2 and 3). The elevation below 1500 m in the Qinling-Huaihe Line region exhibited contributions greater than 90% (Table 2). Additionally, the distribution probability of SARS-CoV-2 decreased with an increase in population density in the upper part of South America, north of the Qinling Mountains and Huai River, and in areas above 1500 m elevation. In these regions, the contribution rate of population density was relatively lower, while the proportions of altitude and land cover were significantly increased. Moreover, when elevation was more than 1500 m on the Pamirs Plateau and up Qinling-Huaihe Line region, land cover also had a quite important impact. The average output result of tenfold cross-validation for COVID-19 indicates that the land cover was significantly influenced in the Northern Hemisphere. The simulation results further emphasized that land cover was the third most important factor influenced the distribution and diffusion of COVID-19 (Fig. 2b). The results reveal that urban areas with a land cover value of 190 exhibit the highest probability of COVID-19 distribution, which also conformed to the actual situation (Figures S3c, S4e, S5a, S6a, S9c, S10ae, S12d, S13ac).

### Influence of climate variables on COVID-19

In regions above 1500 m altitude, the impact of population density decreases, and the impact of climate factors increases (Figure S2). Continuous low-probability predictors for COVID-19 include temperature, incident solar radiation, and rainfall. When the altitude is below 1500 m, Tmax1 (Maximum temperature of January) (SDM-10, SDM-18, and SDM-27), Tmax9 (Maximum temperature of September) (SDM-28), and Bio12 (Annual Precipitation) (SDM-18) were the most important variables influencing the transmission of COVID-19 (Table 2). When the altitude is more than 1500 m, Tmin7 (Minimum temperature of July) (SDM-12), Tmin12 (Minimum temperature of December) (SDM-29), Bio5 (Max Temperature of Warmest Month) (SDM-19), and Temp10 (Mean temperature of October) (SDM-9) were the most important variables influencing the transmission of COVID-19 (Figure S2) (Table 3). The maximum temperature of the warmest month in India, with an elevation above 1500 m, emerged as the most influential variable on the distribution of COVID-19, followed by Tmax3; temperature seasonality was the least influential factor (Table 1). The environmental variables (temperature, solar radiation, and precipitation) predominantly influence the occurrence of COVID-19 during spring and summer near the poles of the northern and southern hemispheres. In contrast, solar radiation in autumn and winter were the main influencing environmental variables in the equatorial region (Figures S3–S14 and Table 1).

### Geographical distribution of COVID-19

**Figure 4:**
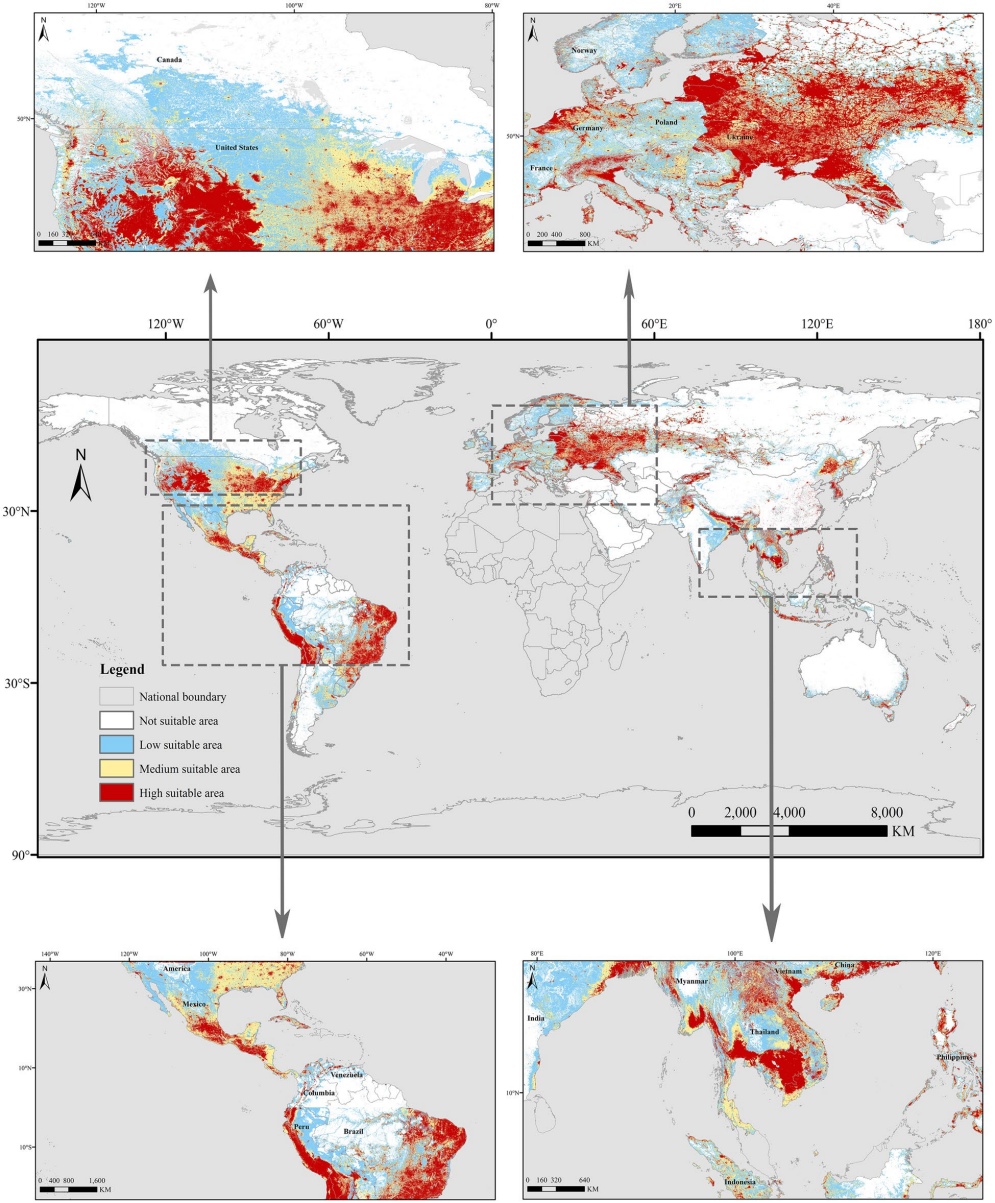
Prediction of the COVID 19 global spatial distribution and potential risk hotspot areas. The map was made in ArcGIS 10.2 using the resulting rasters produced by MaxEnt.

The impact of demographic factors (population density) and environmental variables (elevation, precipitation, incoming solar radiation, and temperature) on the transmission dynamics of SARS-CoV-2 was assessed with the jackknife analysis (Figure S15–S18). The jackknife analysis, a systematic form of re-sampling, repeats the process by leaving out a different value and recalculating the test statistic for each time. The model output was reclassified into four types of potential distributions as follows: not suitable area (0–0.2); low suitable area (0.2–0.4); medium suitable area (0.4–0.6); highly suitable area by ArcGIS 10.2^48,49^. Figure 4 encompasses the global potential distribution mapping of COVID-19, illustrating the comprehensive scope of the virus's potential spread across different regions and locales. The high-risk areas for COVID 19 were located between latitudes 0–50°N and 0–30°S. These include the central and lower parts of North America, concentrated in the northwest and southeast of the United States, as well as central and southern Mexico. In parts of South America, there are western Peru, northern Chile, and eastern Brazil. In the Eurasian continent, the high-risk areas are in Northwest and southern Asia, distributed in southern Myanmar, northern and southern Thailand, northeastern Vietnam, and southern China; Southeast Europe, including all of Ukraine, northwest Germany, western, northern, and southeastern France; and the western part of the Arctic Circle. Ukraine, Belarus, southwestern Russia, northwestern Germany, small areas in southern Guangzhou, southern Harbin, and the entire Changchun region of China also showed high risk. South Korea, Cambodia, southern Myanmar, and southern Vietnam were also classified as high-risk. Additionally, there are high-risk areas in Southeast Oceania, Cuba as a whole, Southeast United Kingdom, Southeast Indonesia, all over the Philippines, southern Japan, and northeastern New Zealand. In North America, most of the central region of the United States and a small portion of the Northeast, as well as small portions of the central northern and southern coastal regions of Mexico, were predicted as medium-risk regions. Central and eastern Ukraine, central and eastern India, northern and Middle eastern Thailand, Hainan, and northeast Harbin in China, and all of Malaysia were also predicted as medium-risk regions. In North America, southern Canada, the northern and southwestern United States, and northern Mexico; in South America, northwest Brazil, Argentina, most of Russia (except the southwest), most of Mongolia, and Australia (except the southern region) were shown as low-risk areas (Fig. 4).

## Discussion

Various studies employed different methods and outcome variables in exploring the influencing factors of the COVID-19 pandemic. The existing literature mainly utilizes a generalized additive model^21^, generalized linear model, Spearman's correlation analysis^50,51^ and Pearson's correlation analysis^52^. Regarding sample selection, the use of national-level samples alone fails to account for the regional variations in weather conditions among countries with large areas and uneven population distribution, such as the United States, China, and Brazil^53^. Moreover, solely selecting geographic areas with confirmed cases as samples for statistical analysis is prone to sample selection bias^53^. However, these methods have certain limitations and may lead to estimation bias since the data often fail to meet the underlying assumptions of the methods. Consequently, they are relatively complex to operate and not user-friendly for beginners. Furthermore, these methods are not suitable for hotspot analysis in regions with incomplete or unreliable reporting. MaxEnt model's ability to handle large datasets and intricate relationships between variables makes it a popular tool for ecological niche modeling and species dispersion. A study on early forecasting of the potential risk zones of COVID-19 in Chinese megacities using the MaxEnt model shows that MaxEnt can meet the timeliness and fine spatial scale requirements for predicting the spread of COVID-19 outbreaks^54^. An analysis using the MaxEnt model to identify the key environmental variables affecting the distribution of the epidemic in Beijing, Shenyang, Dalian, and Shijiazhuang has also demonstrated the efficiency of the model^55^, providing valuable insights for targeted intervention strategies. However, these studies have thus far only focused on a few cities with severe epidemics and have not been comprehensively analyzed. Coro et al. used the MaxEnt model to simulate the global distribution of COVID-19. However, as the study only conducted modeling at the global level, there are significant irrationalities in the evaluation criteria, and the results cannot fully reveal the ecological niche requirements of the novel coronavirus^56^.

Therefore, the MaxEnt model was utilized in this study to assess global COVID-19 data at various spatial scales, which could accurately determine the spatial distribution and main influencing factors of potential infection risk areas at a fine scale of 1 km × 1 km, especially in regions where reporting may be incomplete or unreliable. In addition, this study adopted local scales for modeling in order to avoid data bias caused by excessive phenological differences in the study areas. In niche modeling, the regional scale prediction model offers greater advantages in terms of model accuracy^57,58^. Moreover, the modeling accuracy is ensured by calculating the CV value to process the urban point data with insufficient precision, which provides a method to plot the risk of COVID-19 associated with epidemiological and environmental factors. This approach holds a significant value not only for COVID-19 but also for the research of other infectious diseases.

This study mainly focuses on epidemiological research conducted before the Omicron variant emerged. The transmission speed of the Omicron variant is significantly faster than that of previous variants, and its immune evasion capabilities have been enhanced^59^. Firstly, studying the COVID-19 virus before the emergence of Omicron allows us to gain a more accurate understanding of the original virus's characteristics and how these characteristics affect the spread and prevention of the pandemic. Secondly, epidemiological data from the early stages of the COVID-19 pandemic can better reflect the virus's natural transmission patterns, which is crucial for understanding the mechanism of virus transmission and evaluating the effectiveness of prevention and control strategies. Successful early warning is crucial for containing the epidemic in its early stages before it escalates into a large-scale outbreak^60^.

Currently, the AUC method is considered the best criterion for assessing the success of presence/absence data models^45^. An AUC value above 0.8 indicates a good model, while an AUC close to 1 signifies excellent performance^61^. Sensitivity is defined as the proportion of test localities correctly predicted to be present (1–extrinsic omission rate). The quantity (1–specificity) equals the proportion of all map pixels predicted to have suitable conditions for the species^62^. An ideal model demonstrates a true positive rate (sensitivity) close to 1 and a false positive rate close to 0 (1–specificity). Most of our models exhibit a positive rate close to 1, indicating high accuracy. This demonstrates the accuracy and reliability of the modeling results. Additionally, a VIF value below 10 indicates low and acceptable multicollinearity^63^. This signifies that the MaxEnt model, having achieved a high level of performance, is suitable for simulating COVID-19 risk areas globally, thus enabling early forecasting of potential infection risks.

In our study, among all 31 SDM models, 25 models were significantly influenced by population density. The results indicate that population density emerged as the most influential variable that affects the distribution of SARS-CoV-2 (Fig. 2b), aligning with other studies that highlight its significance in the spread of SARS-CoV-2 using the MaxEnt model^54^. Numerous reports on the distribution of COVID-19 by other methodological investigations consistently validate our research findings, emphasizing the coherence and reliability of our study in this particular context^64–69^. As a respiratory virus, SARS-CoV-2 is mainly transmitted through respiratory droplets; therefore, population density plays an important role in the spread of COVID-19^70^. It is more difficult to maintain a safe distance between people in places with high population density, which increases the possibility of virus transmission^70^. The interconnectedness of cities worldwide and their intricate ecosystems facilitate the transmission of the virus among individuals, while the complexities stemming from urbanization and social cohesion exacerbate efforts to control the global pandemic^71^. Transmission was more severe in densely populated communities, fostering the spread of SARS-CoV-2 to varying degrees^72,73^. Identifying crowded places in time (local residents or densely populated floating population) can serve as one of the key measures to cut off the transmission^74^. A research suggests that total import and export of provinces has a high association with confirmed cases over time^37^. International trade emerged as a comprehensive indicator encompassing population density, human mobility, and economic dynamism, thus highlighting the significance of demographic factors^75^.

The model showed meteorological factors can also be considered an influencing factor for the COVID-19 transmission of pathogens. Our results align with recent worldwide studies on the effect of climate on the spread of the COVID-19, which have shown that temperature and humidity were not crucial factors in the COVID-19 transmission^76^. There was a nonlinear relationship between ambient temperature and morbidity. We found that the threshold was around 10–25 °C, which is similar to other global studies^70,77^. Recognizing a specific temperature threshold can serve as a triggering factor for early warning of COVID-19^78^. Meteorological factors can affect the transmission of the virus in two different ways, such as from an epidemiological and behavioral perspective. The viability of infectious viruses depends on environmental factors such as temperature and humidity^79,80^, with high temperatures damaging the virus's lipid envelope^22,81^. Higher temperatures severely impair the survival ability of the SARS coronavirus^82^. While low temperature and low humidity enhance the stability of droplet transmission in the nasal mucosa. In behavioral perspective, weather can alter levels of activity, social distance, and social gathering locations, thereby influencing the spread of the virus among individuals^83^. An increase in temperature range between 10 and 25 °C corresponded to a higher probability of SARS-CoV-2 transmission. However, a significant increase in temperature above 25 °C reduced its probability. That may be due to the fact that moderate temperatures increase human activity. Additionally, considering that the transmission of coronavirus was similar to influenza, influenza virus was more transmissible at lower temperatures because cold weather can weaken the host's immune system, thereby increasing infection susceptibility. There is no conclusive evidence indicating that the number of COVID-19 cases decreases as the weather warms up^84^, which offers valuable insights for policymakers and the general public. Lower temperatures enhance the stability of the viral lipid envelope, thereby extending the survival and transmission capabilities of SARS-CoV-2^77^. Additionally, our research revealed a negative correlation between temperature and the probability of COVID-19 transmission in hot regions. This may be due to the fact that people in hot regions tend to reduce their outdoor activities due to unfavorable climatic conditions. In our study, the importance of relative humidity ranks last among all meteorological variables, indicating that relative humidity may be a secondary determinant of local transmission of COVID-19. Similar findings have also been concluded in epidemiological studies^70^. The results show that in continental areas, the impact of relative humidity on the spread of COVID-19 exhibits a "U" shape, which is consistent with other studies^70^. In island countries such as Japan and the Philippines, there is a positive correlation between relative humidity and the spread of COVID-19. Some studies have found that relatively high humidity environments can reduce individuals' cognitive abilities, making it difficult for them to think clearly and reducing their alertness^85^. We hypothesized that this could affect people's prevention efforts against COVID-19. Therefore, the formulation of epidemic prevention and control measures should take into account the actual conditions of each region.

Several recent studies argue that land cover may be a critical factor in the COVID-19 pandemic^41,86^. MaxEnt results indicated that land cover, in particular, significantly impacts the spread of COVID-19 (Fig. 2b). The results indicated that when the land use type is urban, the probability of COVID-19 outbreak is higher as depicted in Figure S3c and Figure S4d. Urban areas have higher human mobility, thus resulting in a higher population density^87^. Land cover played a synergetic role in affecting human populations and the spread of terrestrial species^88–90^. More and more people are living or migrating in densely populated residential, commercial, and administrative areas, which increases the likelihood of contracting the coronavirus^38^. An increase in natural land cover in living environments might not directly prevent the spread of COVID-19, but it improves public health status. In other words, with more natural land cover, people may have fewer clinical factors associated with a high risk of death from being infected by COVID-19^91^. The study indicates that natural land cover could reduce COVID-19 prevalence and mortality in both the long and short terms^41^.

This study demonstrated that the effect of altitude on mortality in COVID-19 exhibited an opposite result, which is consistent with the findings of other studies^43,73,92^. Several possible explanations have been proposed for the protective effect of altitude. First, in high-altitude environments, chronic hypoxia significantly reduces the expression of ACE2 in pulmonary arterial smooth muscle cells, thus decreasing the risk of COVID-19 infection^93,94^. Second, it is also possible that the levels of hypoxia encountered may optimize cellular oxygenation, antioxidant systems and mitochondrial performance at the alveolar level by populations in higher altitudes with the potential to resist SARS-CoV-2 related complications^95^. Third, studies have shown that due to the lower density of air and greater distance between molecules at high-altitude, which may reduce the size of the airborne virus inoculum and the probability of dissemination between people^96^. Finally, Solar radiation is typically stronger in high-altitude areas than in low-altitude regions. The model showed an increase in solar radiation within a certain range leads to a significant decrease in the daily number of cases, consistent with laboratory studies showing that UV light can deactivate viruses in the air and on surfaces^97^. Our findings are reinforced by multiple studies^70,98^. Excessive solar radiation, however, can limit potential human activities^97^. Additionally, a meta-analysis showed that 41% of COVID-19 patients suffered from vitamin D deficiency and 42% had an insufficient vitamin D level^98^. The regular exposure to sunlight can facilitate the production of vitamin D, thereby strengthening human immune system and resilience against viral infections^70^.

This study introduces a multi-factor risk prediction system and emphasizes the important role of different variables in disease transmission for global health strategies. This finding enhances our understanding of COVID-19 transmission dynamics, emphasizing the significant influence of demographic, geographical, and environmental factors. The findings have implications for public health strategies and emphasize the need for comprehensive, localized modeling to effectively address the global challenges posed by infectious diseases like COVID-19. Furthermore, understanding the reasons and influencing factors behind the rapid spread of the disease and dividing risk distribution areas may identify the key areas for disease prevention and control. We should develop prevention and control plans that can be implemented scientifically and effectively based on the principles of epidemic transmission, ensuring the main aspects of both prevention and control. Prompt and efficient execution of these tasks can lead to significant savings in manpower and material resources. Overall, the MaxEnt model can be used as an early prediction tool to identify the risk distribution range of COVID-19, especially hotspots, high-risk areas, and transmission areas, and potential infection risk areas for COVID-19 at a fine scale, considering factors such as population density, meteorological factors, altitude, solar radiation, and land cover. Notably, population density emerges as the most significant predictor. Meteorological factors and land cover types significantly impact the spread of COVID-19, while solar radiation and altitude are negatively associated with the number of COVID-19 cases. Additionally, temperature has significant effects on the spread of COVID-19, while precipitation has the least impact.

Our study has three major limitations. First, in order to ensure the accuracy of the model, certain regions like Africa had to be excluded due to insufficient data reliability. Missingness in data may indicate potential problems in data pre-processing and may have influenced the results. Secondly, since many cities have implemented corresponding intervention measures, spatial analysis models can be introduced to identify potential COVID-19 infection risk areas in different regions by combining prevention and control policies. By comparing these risk areas with the ones from this study, the effectiveness of prevention and control strategies can be further evaluated. Thirdly, we have only evaluated the influencing factors of early COVID-19, and long-term data can be included for further verification and comparison in the future.

## Methods

### Differentiation of prediction areas

We conducted an analysis of the epidemiological patterns of COVID-19 worldwide based on the COVID-19 occurrence reports from every region, except the Africa region, due to the unavailability of official data. The WWF (World Wide Fund for Nature) global ecological zoning, established for natural conservation purposes (Eco-regions), was adopted as the basic framework for the global ecological geographic zoning knowledge base in this article^99^. The analysis was performed separately for six island countries, i.e., Japan, Indonesia, New Zealand, the United Kingdom, Ireland, and Cuba. The epidemiological characteristics of SARS-CoV-2 were accurately analyzed in the above-mentioned landscapes. Briefly, the regional study on the global continents was conducted according to the altitude, topography, and climate characteristics of each continent, combined with the global temperature zone^100–104^. Subsequently, MaxEnt was applied for each region separately (Fig. 1).

### COVID-19 occurrence records and processing

The early COVID-19-infected cases, spanning from January 1, 2020, to October 30, 2021, across 173 countries, were sourced from WHO (World Health Organization)^10^. To enhance the accuracy of the species distribution model (SDM), a meticulously screened process was applied to the COVID-19 point data. Excluding cases from countries or regions lacking transmission results. Furthermore, to address potential data shortages at the local level and to enhance the accuracy of our analysis, we refined the COVID-19 data necessary for the MaxEnt model and employed it for guiding our variable selection. We calculated the coefficient of variation values (CV) by utilizing 67 climate variables, which reflect the degree of dispersion between data points^105^. This method serves to quantify the data within the dataset. To conduct a high-precision analysis, a grid size of 1 km^2^ within each city was employed. The homology of the city was acceptable, given that the CV values of all variables were less than 15%^105,106^. The geometric center of the city was retained for subsequent MaxEnt modeling. We indicated the training and test datasets in Figure S1.

### Processing of environmental variables

Environmental predictor variables, including climate, terrain, vegetation, and human impact, were generated for COVID-19 modeling. The current forecasting data was collected from the CHELSA database (Table 4)^107,108^. The incoming solar radiation (ISR) values were calculated at 30-min intervals and aggregated per growing season. The seasonal category of each research area was integrated from official data from each country, survey reports, and the website of the global seasons division (https://seasonsyear.com/). All spatial data preprocessing and calculations were done with standard operations in ArcGIS 10.2 and were projected in UTM-WGS-1984 with standard settings or resampling to 30 arc-seconds^44,45,109^.

**Table 4:** The environmental predictor variables of layers, sources, categories and variables/proxy used in modelling of COVID-19 distribution.

Layers	Source	Value/categories	Variable/proxy
Climate^a^			
Monthly P (prec1-12)	Ibid	0 to 1201 mm/month	Precipitation
Monthly mean T (temp1-12)	Ibid	− 54.9 to 39.2 °C	Mean Temperature
Monthly min T (tmin1-12)	Ibid	− 56.5 to 32.3 °C	Minimum Temperature
Monthly max T (tmax1-12)	Ibid	− 53.2 to 47.3 °C	Maximum Temperature
Bioclimatic (bio1-19)	Ibid		Annual trends, seasonality, extreme or limiting environmental variables
Terrain^b^			
Elevation	ASTER-GDEM	− 328 to 4739 m a.s.l	Climbing distance
ISR-spring	Ibid	62.4 to 206.8 wh/m^2^	Topo-climate
ISR-summer	Ibid	35.5 to 104.2 wh/m^2^	Topo-climate
ISR-autumn	Ibid	64.1 to 218.2 wh/m^2^	Topo-climate
ISR-winter	Ibid	58.1 to 184.2 wh/m^2^	Topo-climate
Vegetation			
Land cover^c^	ESA	Cropland (3), Herbaceous, Tree (9), Shrubland (3), Grassland, Urban areas, Bare areas (2), Mosaic shrub & herbaceous cover, Water bodies, Permanent snow, and ice	Human activity venues
Human impact			
Human population^d^	WorldPop	0 to 1,202.6 ind/km^2^	Human-Animal interaction

^a^T = temperature; P = precipitation. Source: http://chelsa-climate.org/

^b^Source: http://www.gscloud.cn/ ; ISR = Incoming Solar Radiation.

^c^Land cover: Cropland, Herbaceous, Tree, Shrubland, Grassland, Urban areas, Bare areas, Water bodies and Permanent snow and ice https://maps.elie.ucl.ac.be/CCI/viewer/

^d^Source: https://www.worldpop.org/

### COVID-19 distribution modeling and evaluation

The MaxEnt model stands out as one of the best-performing specialty distribution modeling techniques for analyzing occurrence data. Consequently, we employed the MaxEnt model to predict the future distribution of COVID-19 infection using case occurrence data^110^. This model developed the ecological niche models by employing a machine-learning approach, combining COVID-19 case occurrence data with environmental variables. To explore the risk situation of SARS-CoV-2, the MaxEnt model was applied to the spatial distribution model building. The areas of interest were categorized into those below and above 1500 m asl, according to the elevation standard of the highland climate^45,111^. Spatial autocorrelation was minimized by filtering all recorded COVID-19 locations data using the SDM Toolbox v1.1c in ArcGIS 10.2^109^. Principal component analysis (PCA) and multicollinearity were addressed by excluding factors through variance inflation factor (VIF) analysis^63,112^. The filtered COVID-19 location and predictors served as input data for constructing the COVID-19 model using the MaxEnt algorithm. We divided the selected occurrence records into 70% training and 30% testing portions to build and validate the models based on 10 bootstrap replicates. For the remaining parameters, we maintained the default settings in the pilot study. The final COVID-19 predicted risk maps for low-elevation and high-elevation areas were overlaid using the fuzzy overlay. The Jenks natural break optimization method was employed to classify the model output with smoothing and visualize high-risk areas^107,113^. The relative contribution of predictors for modeling was evaluated through the jackknife test and variable response curve. The accuracy of the model was assessed by the area under the receiver operating characteristic (ROC) curve^114^.

### Supplementary Information

Supplementary Information.

## Data Availability

The environmental predictor variables have been deposited in the CHELSA (http://chelsa-climate.org/), the terrain predictor variables have been deposited in the Geospatial Data Cloud (http://www.gscloud.cn/), the population destiny was download in (https://www.worldpop.org/), Land cover was download in ESA (https://maps.elie.ucl.ac.be/CCI/viewer/). Materials supporting the findings of this study are available from the corresponding authors upon request.
