# Supplementary material for: Assessing eco-geographic influences on COVID-19 transmission: a global analysis
Source: Sci Rep. 2024 May 22;14:11728. doi: 10.1038/s41598-024-62300-y (PMC11111805; doi:10.1038/s41598-024-62300-y)
Supplement: Supplementary file 1 — Supplementary Information. [file 41598_2024_62300_MOESM1_ESM.pdf]

Supplementary

Assessing Eco-Geographic Influences on COVID-19 transmission: A global analysis

Authors： Jing Pan[1,2], Arivizhivendhan Kannan Villalan[1,2], GuanYing Ni[3], RenNa Wu[3], ShiFeng Sui[4], XiaoDong Wu[5*], XiaoLong Wang[1,2*]

Affinity：

[1]Key Laboratory for Wildlife Diseases and Bio-Security Management of Heilongjiang Province, Harbin 150040, Heilongjiang province, P. R. China.
[2]College of Wildlife and Protected Area, Northeast Forestry University, Harbin 150040, Heilongjiang province, P. R. China.

[3]HaiXi Animal Disease Control Center，Delingha 817099，Qinghai province, P. R. China.

[4]Zhaoyuan Forest Resources Monitoring and Protection Service Center, Zhaoyuan 265400, Shandong province, P. R. China.
[5]China Animal Health and Epidemiology Center, Qingdao 266032, Shandong province, P. R. China.
[*]Corresponding Author: XiaoDong Wu. Email: wuxiaodong@cahec.cn.
[*]Corresponding author: XiaoLong Wang. Email: nefuwxl@hotmail.com; wxlhrb@nefu.edu.cn.

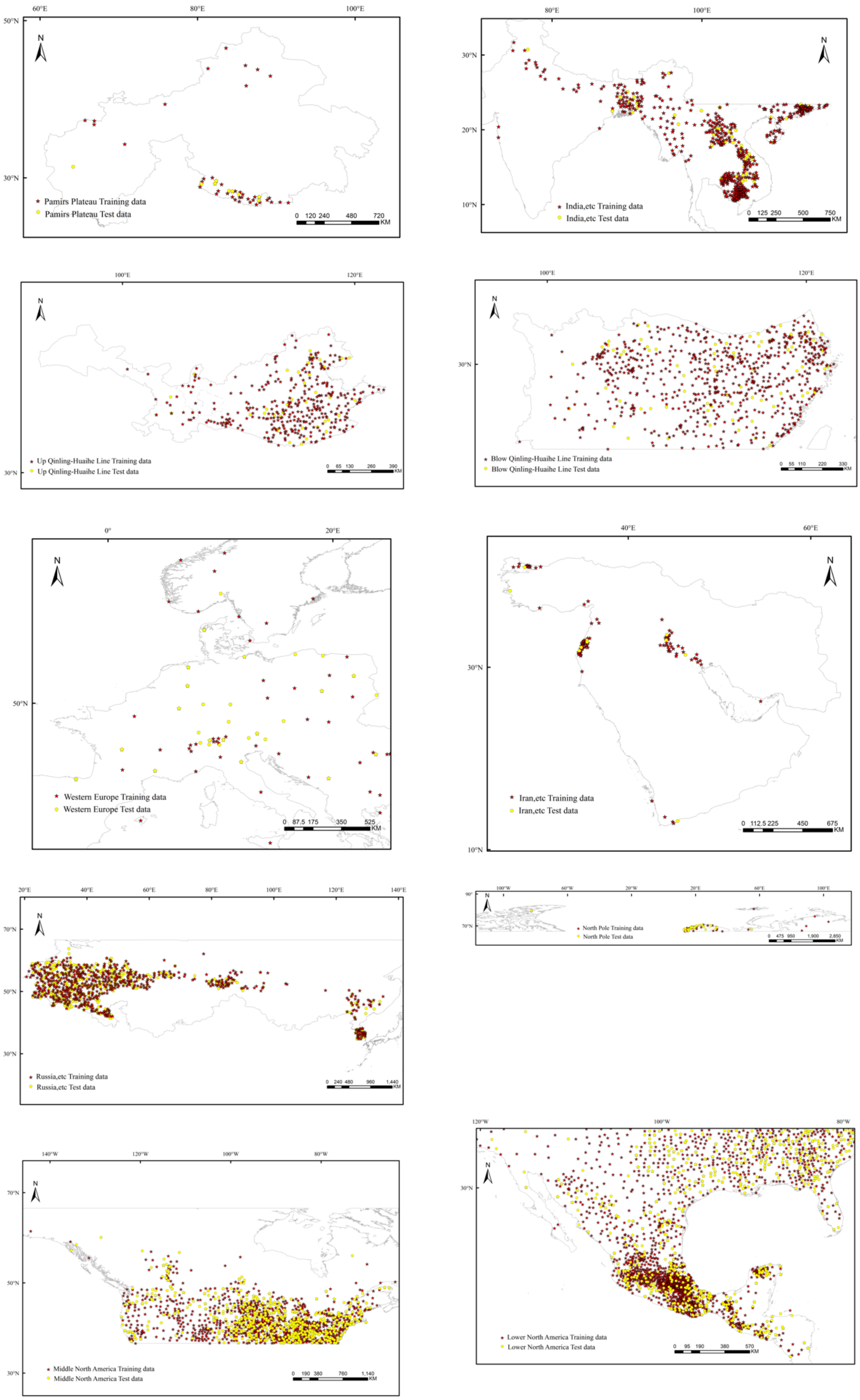

Pamirs Plateau Training data
Pamirs Plateau Test data
India,etc Training data
India,etc Test data
Up Qinling-Huaihe Line Training data
Up Qinling-Huaihe Line Test data
Blow Qinling-Huaihe Line Training data
Blow Qinling-Huaihe Line Test data
Western Europe Training data
Western Europe Test data
Iran,etc Training data
Iran,etc Test data
Russia,etc Training data
Russia,etc Test data
North Pole Training data
North Pole Test data
Middle North America Training data
Middle North America Test data
Lower North America Training data
Lower North America Test data

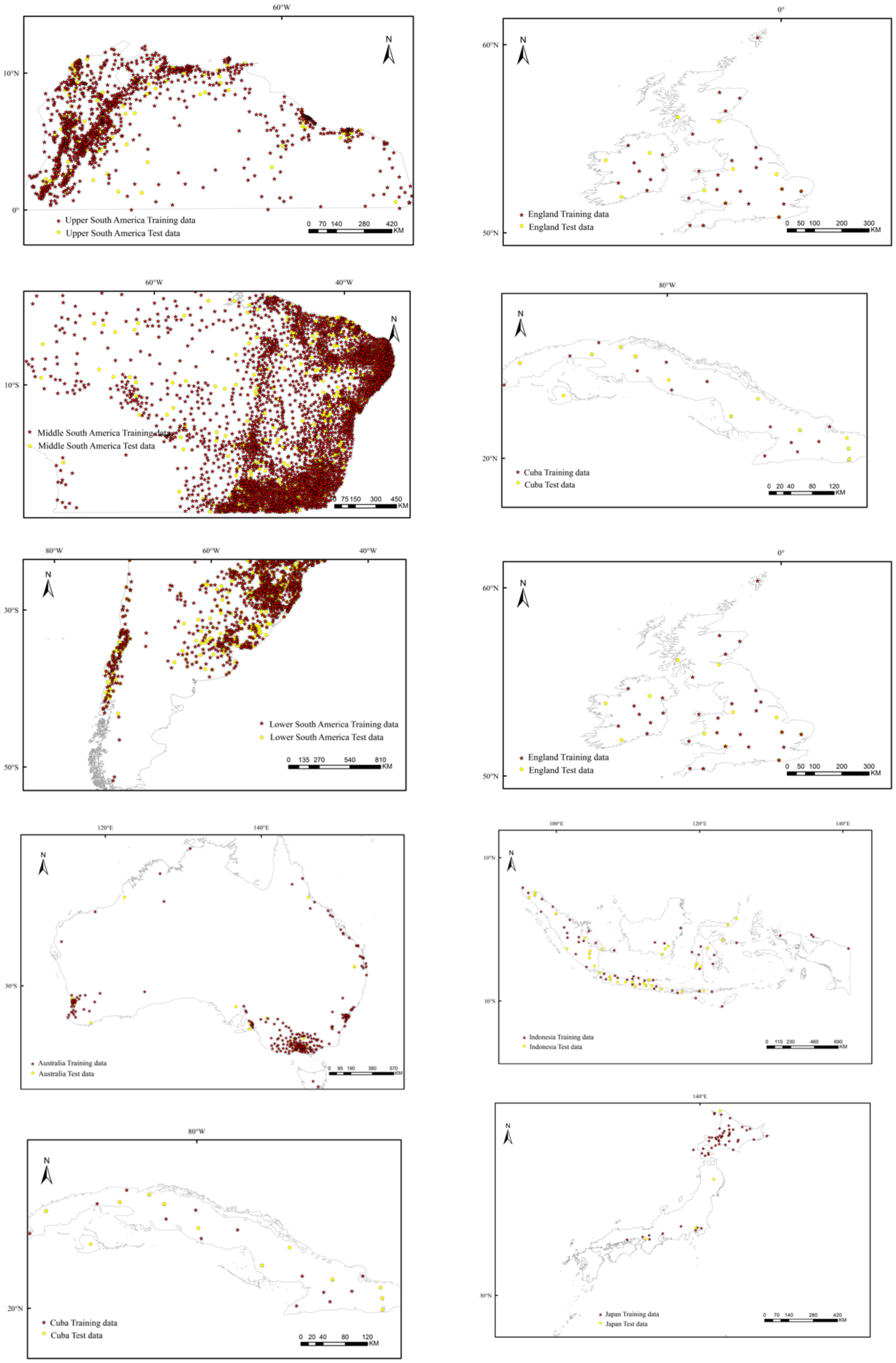

Figure S1. The spatial distribution of the training and test datasets of 20 areas. The map is only used for data display, which is a schematic line illustrating the relative position of each country and should not be re-used or misinterpreted for any political reason.

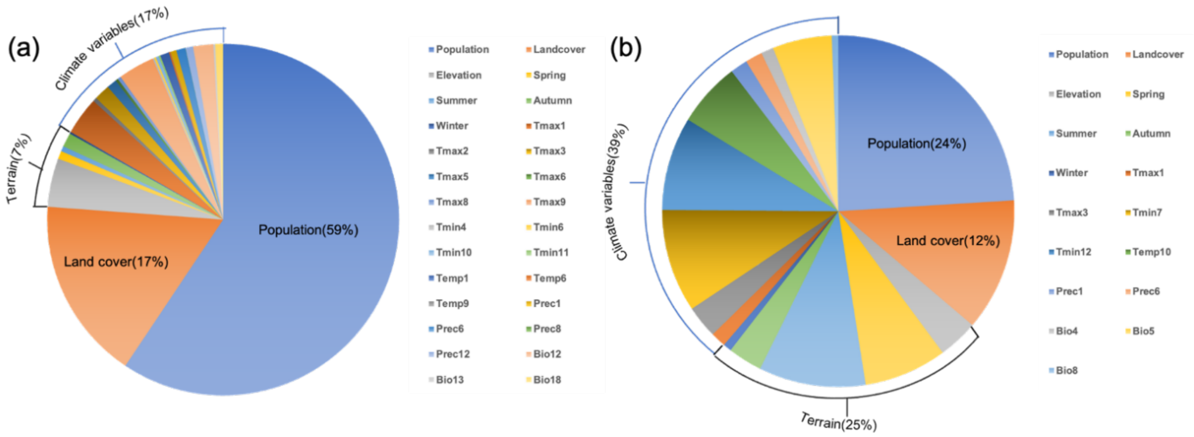

Figure S2. The percentage contributions of predictor variables in (a) altitude blow than 1500m and (b) altitude above than 1500m.

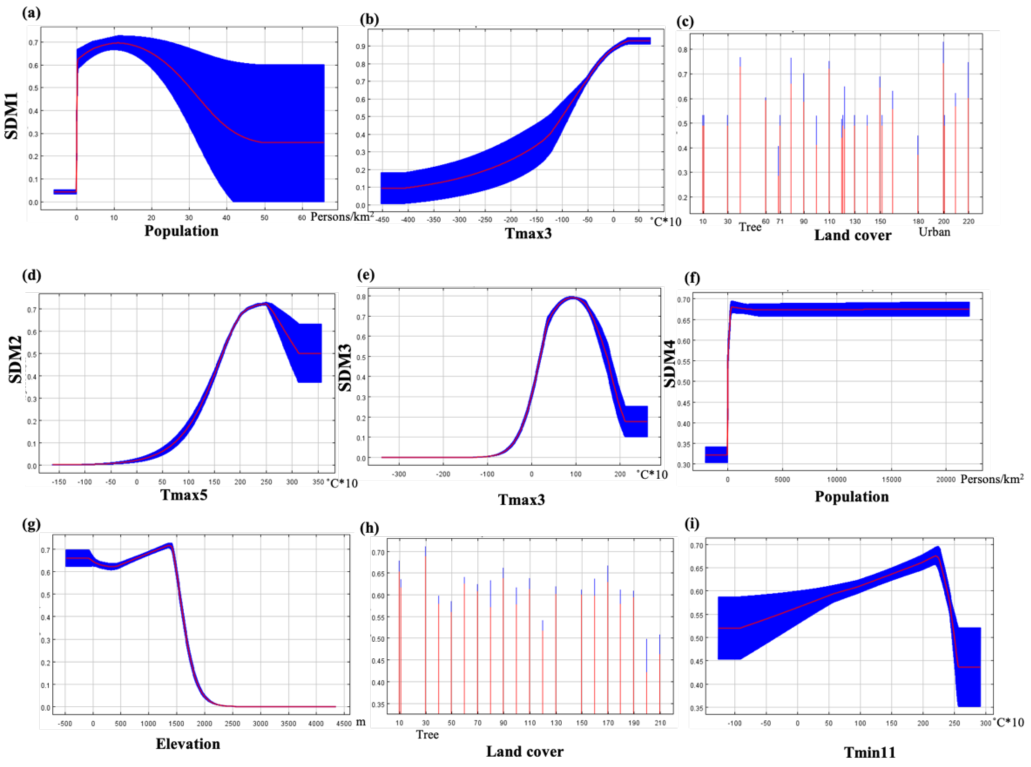

Figure S3. Analysis of COVID-19 distribution models' response curves with with a contribution rate less than 80% in models were (a-c) SDM1, (d) SDM2, (e) SDM3, (f-i) SDM4.

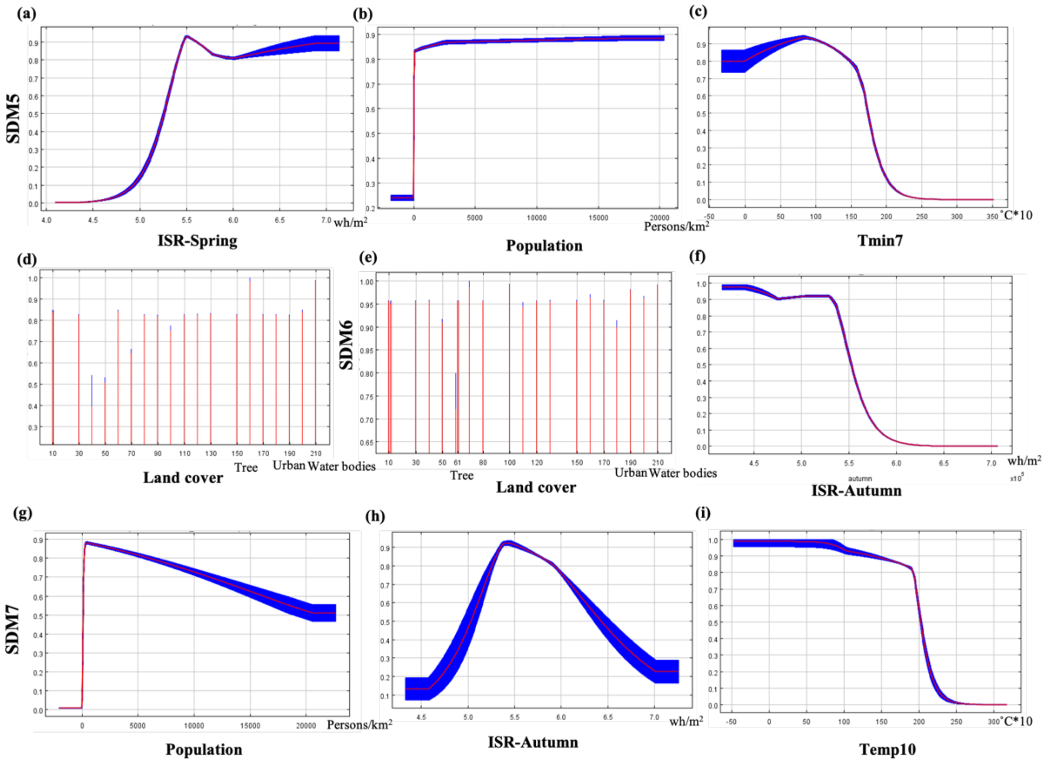

Figure S4. Analysis of COVID-19 distribution models' response curves with with a contribution rate less than 80% in models were (a-d) SDM5, (e,f) SDM6 and (g-i) SDM7.

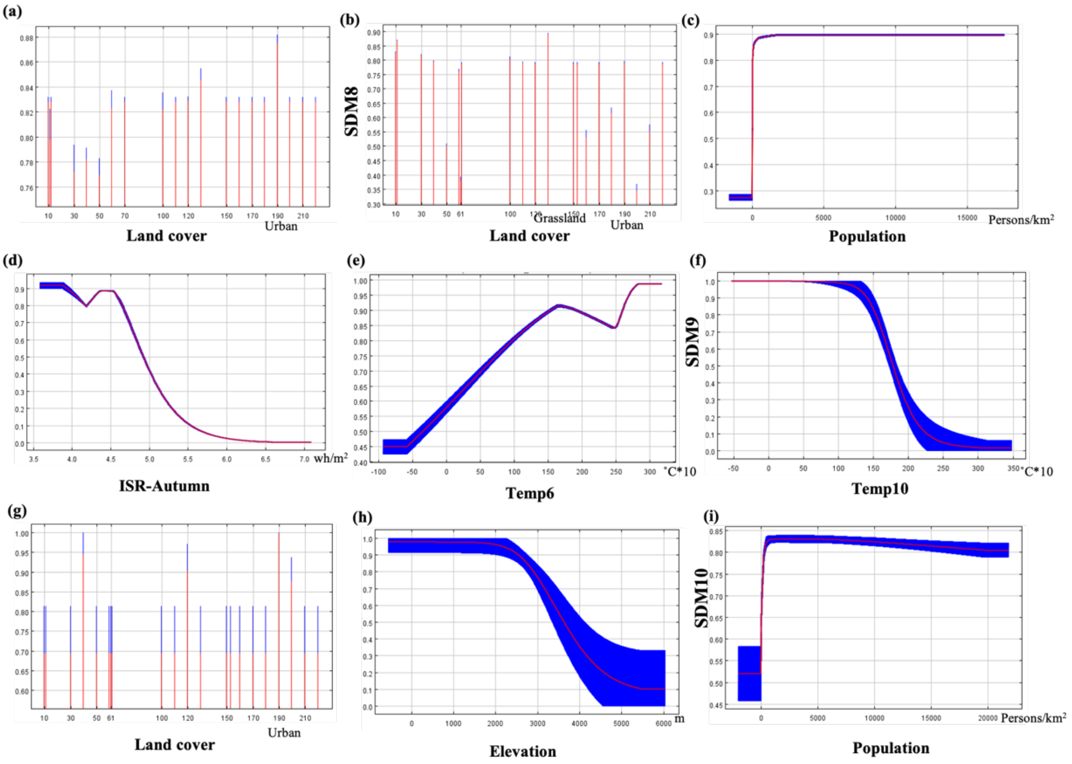

Figure S5. Analysis of COVID-19 distribution models' response curves with with a contribution rate less than 80% in models were (a) SDM7, (b-e) SDM8, (f-h) SDM9 and (i) SDM10.

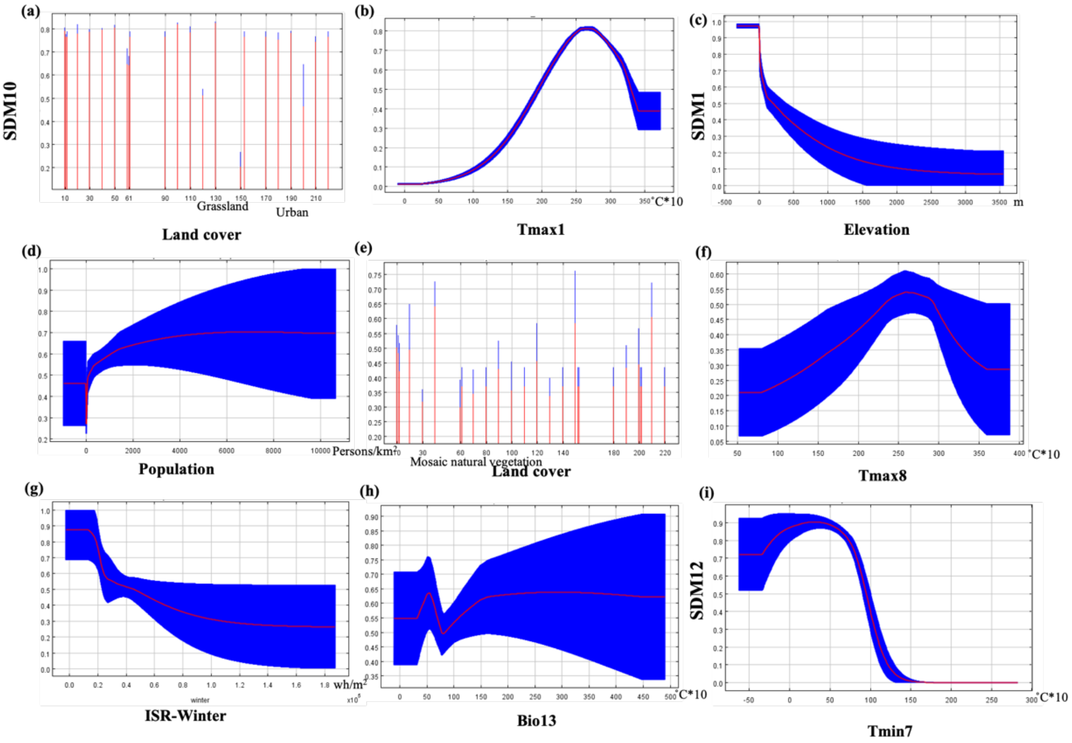

Figure S6. Analysis of COVID-19 distribution models' response curves with with a contribution rate less than 80% in models were(a,b) SDM10, (c-h) was SDM11 and (i) SDM12.

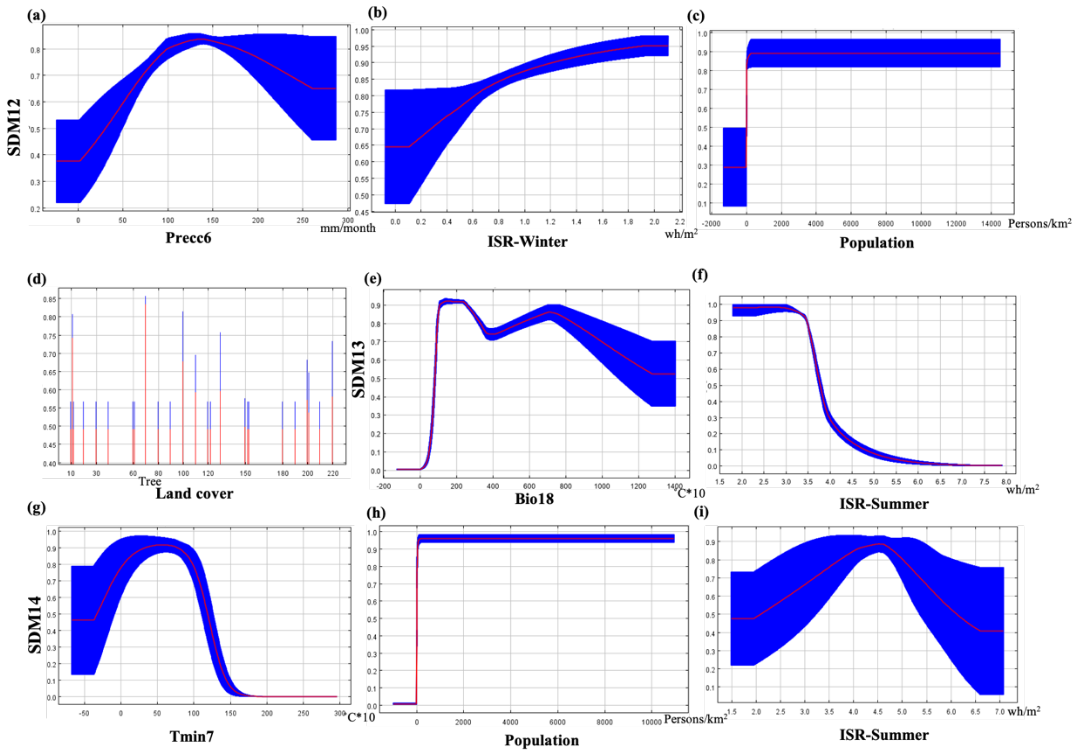

Figure S7. Analysis of COVID-19 distribution models' response curves with with a contribution rate less than 80% in models were (a-d) were for SDM12, (e, f) SDM13 and (g-i) SDM14.

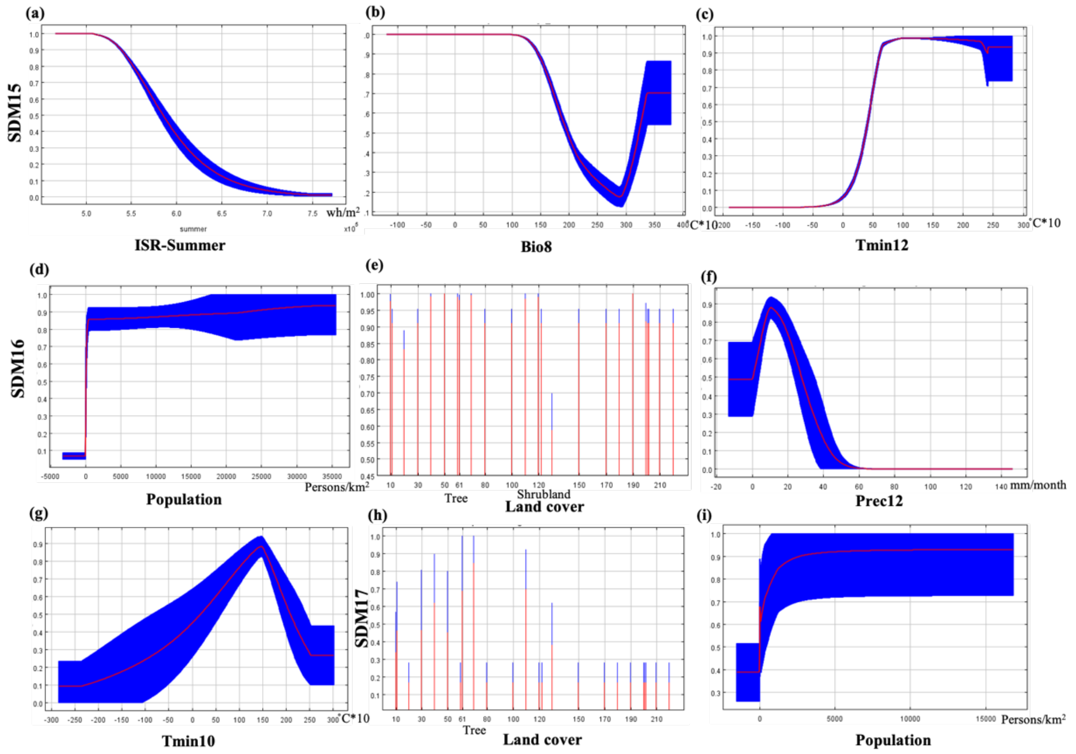

Figure S8. Analysis of COVID-19 distribution models' response curves with with a contribution rate less than 80% in models were (a-c) were for SDM15, (d-g) was SDM16, (h,i) was SDM17.

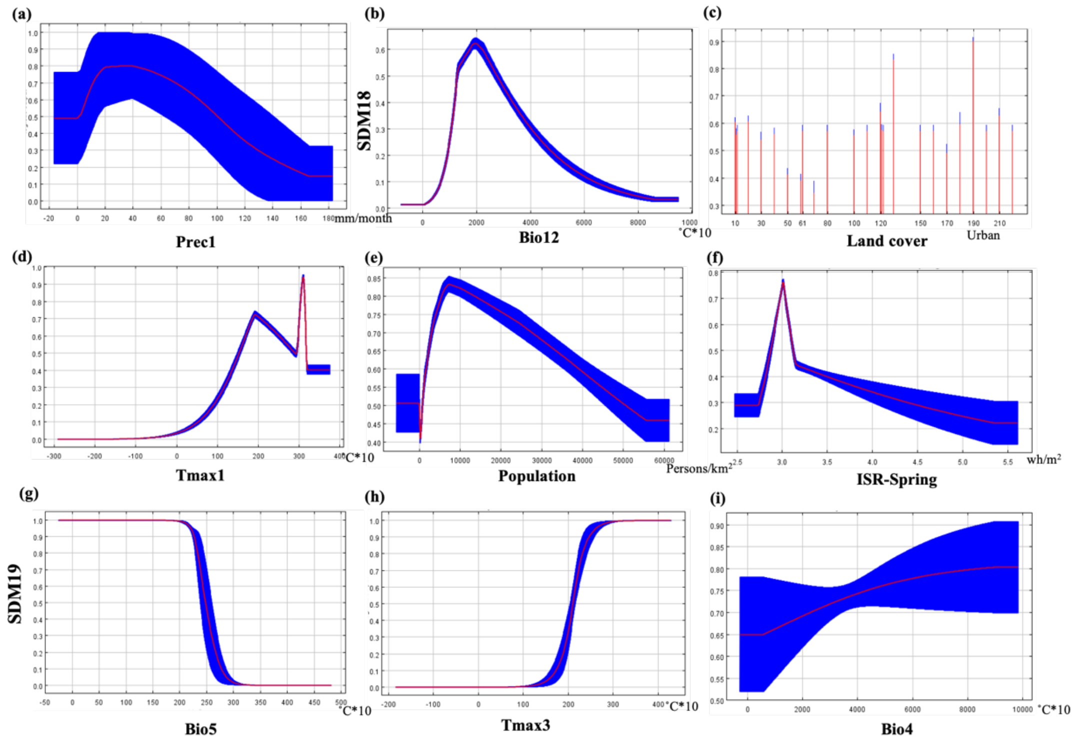

Figure S9. Analysis of COVID-19 distribution models' response curves with with a contribution rate less than 80% in models were (a) SDM17, (b-f) SDM18, (g-i) SDM19.

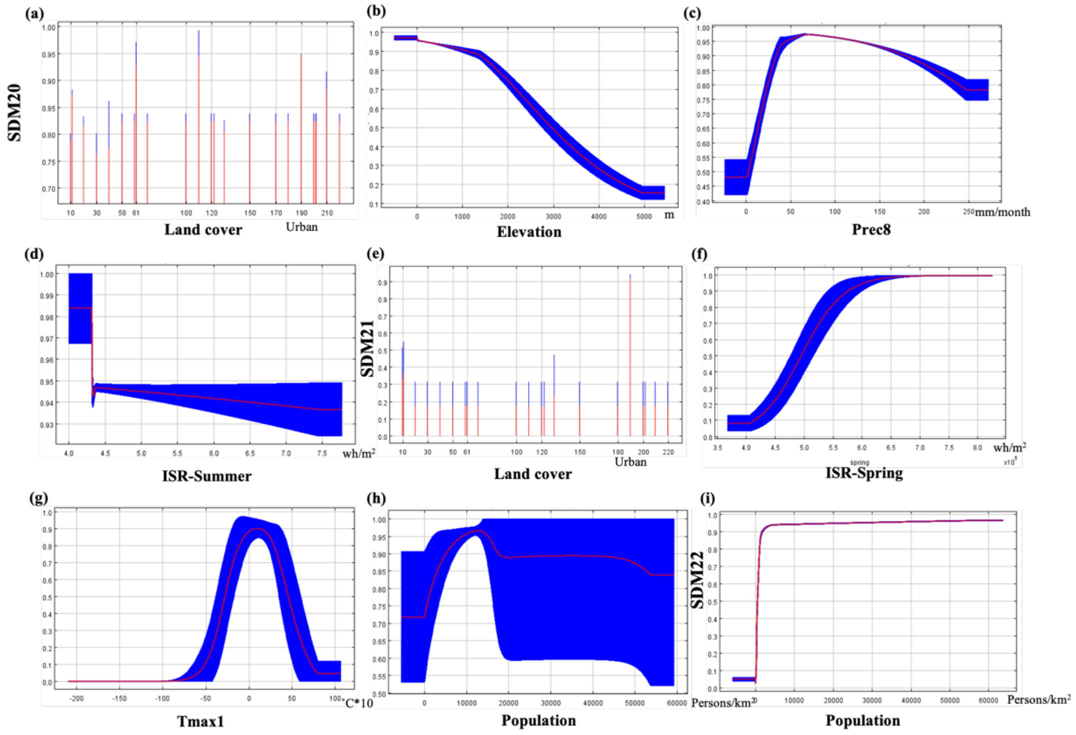

Figure S10. Analysis of COVID-19 distribution models' response curves with with a contribution rate less than 80% in models were (a-d) SDM20, (e-h) SDM21 and (i) SDM22.

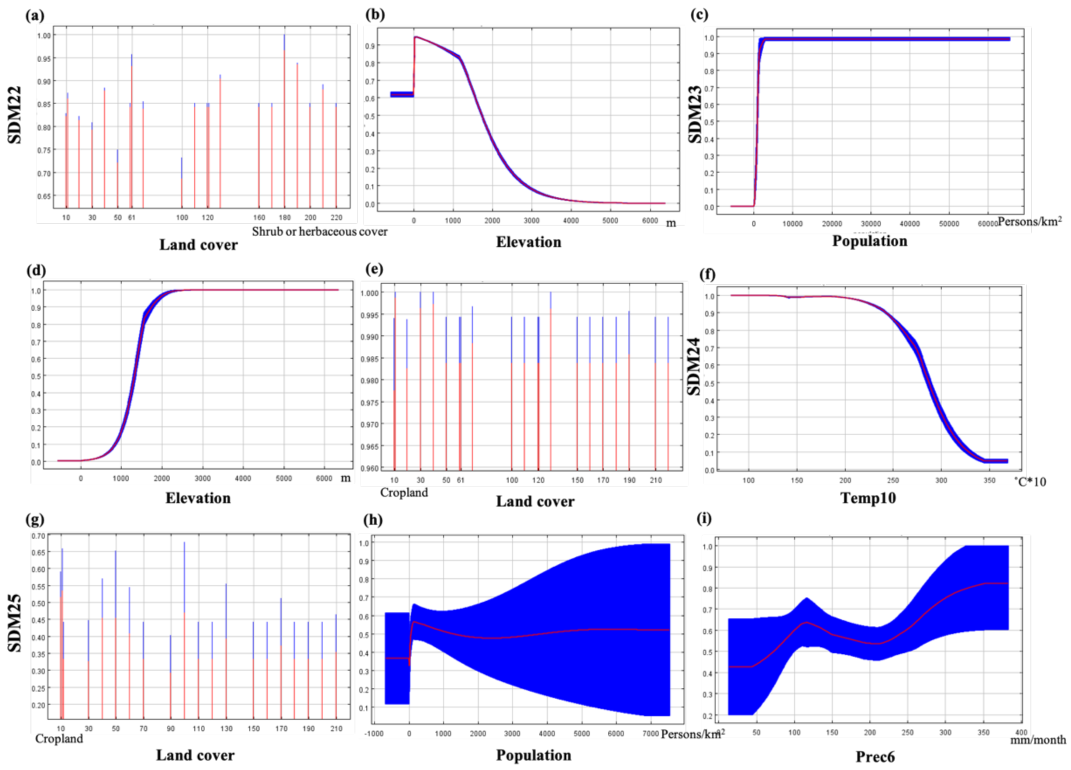

Figure S11. Analysis of COVID-19 distribution models' response curves with with a contribution rate less than 80% in models were (a-b) SDM22, (c-e) SDM23, (f) SDM24 and (g-i) SDM25.

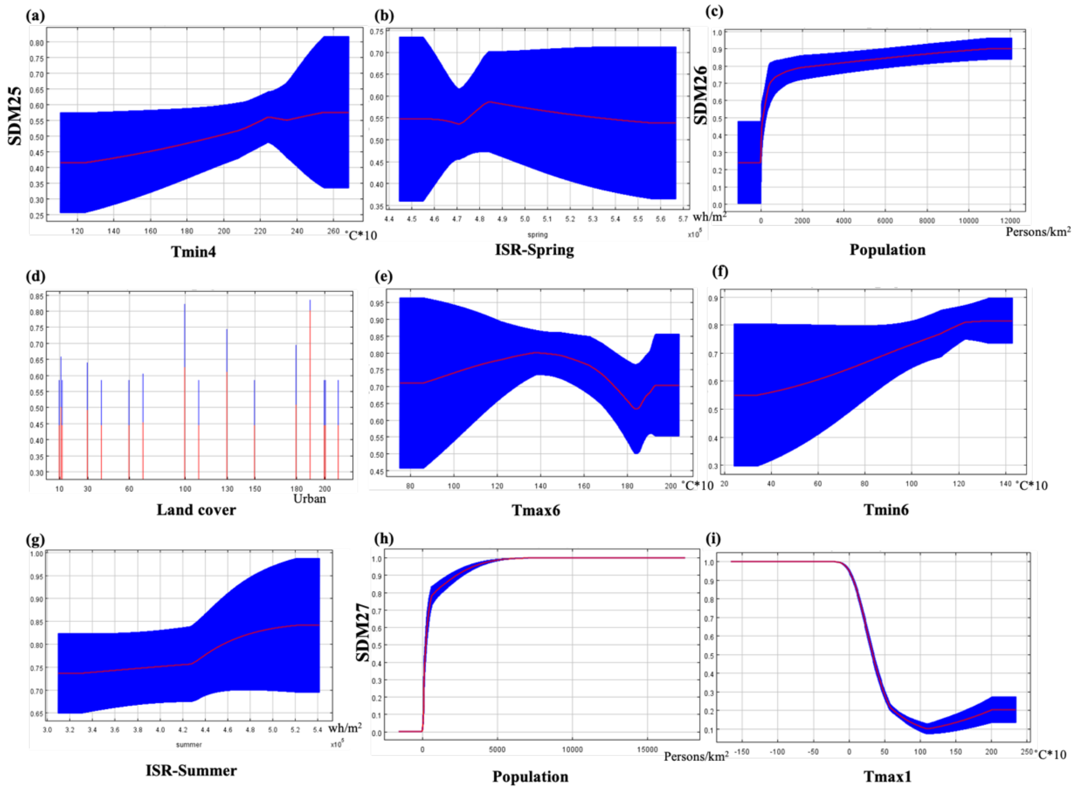

Figure S12. Analysis of COVID-19 distribution models' response curves with with a contribution rate less than 80% in models were (a,b) SDM25, (c-g) SDM26 and (h, i) SDM27.

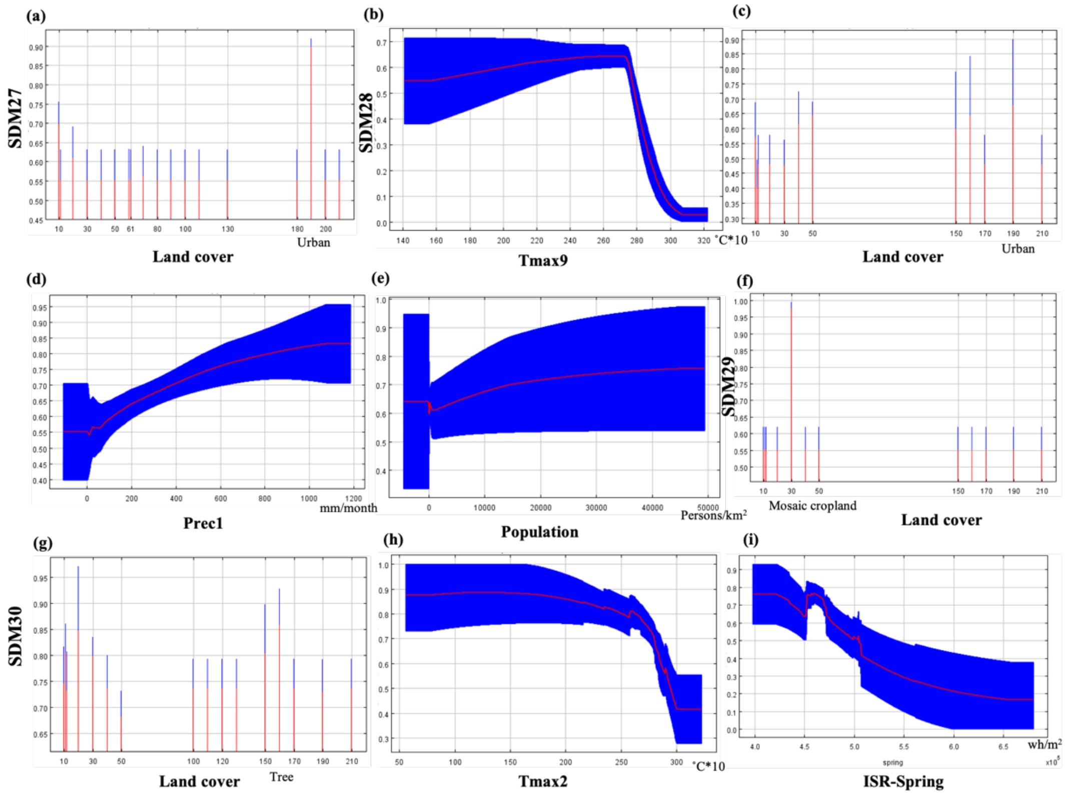

Figure S13. Analysis of COVID-19 distribution models' response curves with with a contribution rate less than 80% in models were (a) SDM27, (b-e) SDM28, (f) SDM29 and (e-i) SDM30.

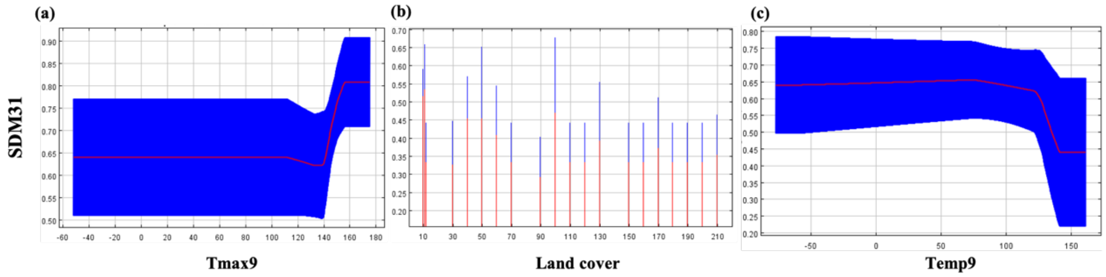

Figure S14. Analysis of COVID-19 distribution models' response curves with with a contribution rate less than 80% in 31 SDM31.

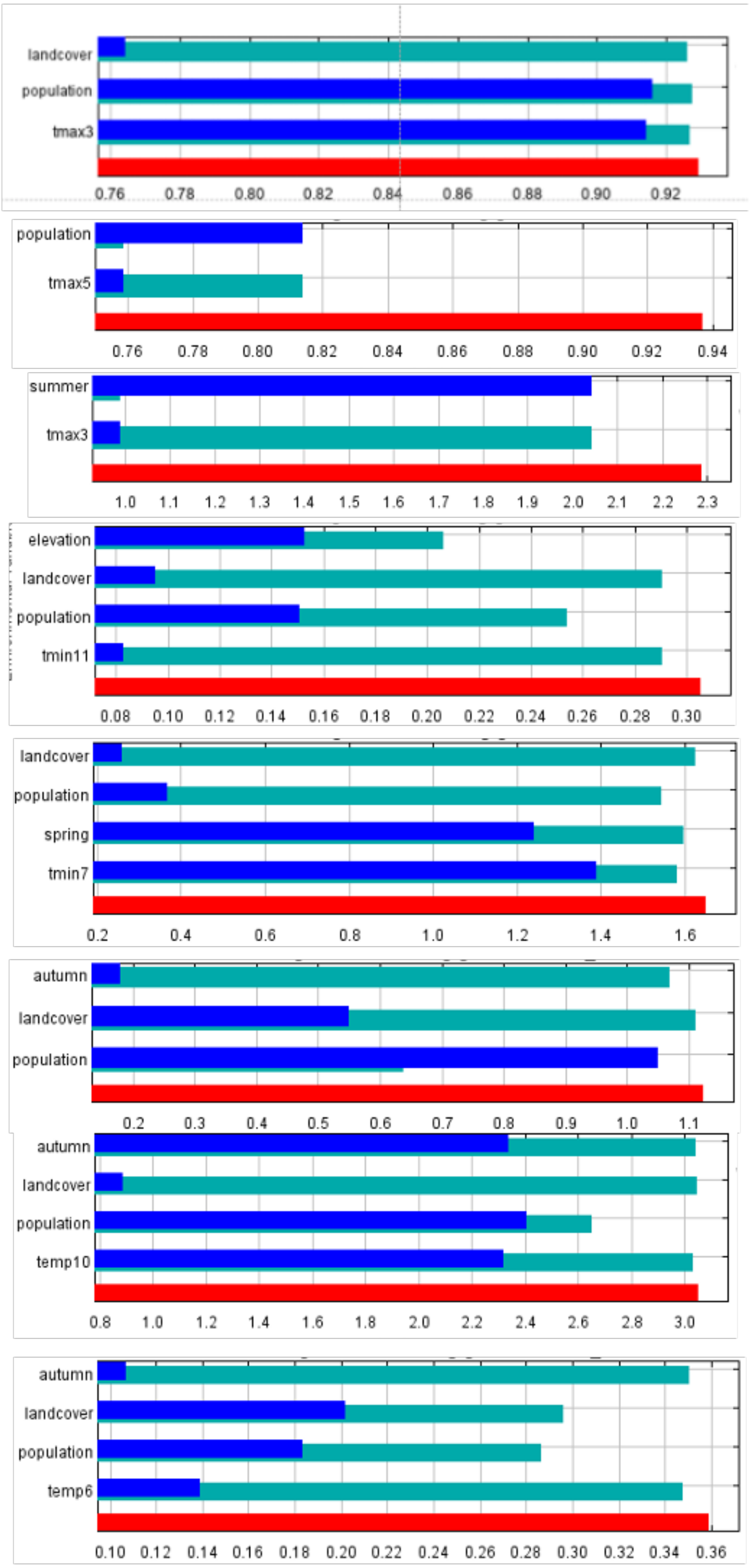

Figure S15. The Jackknife of SDM1-8

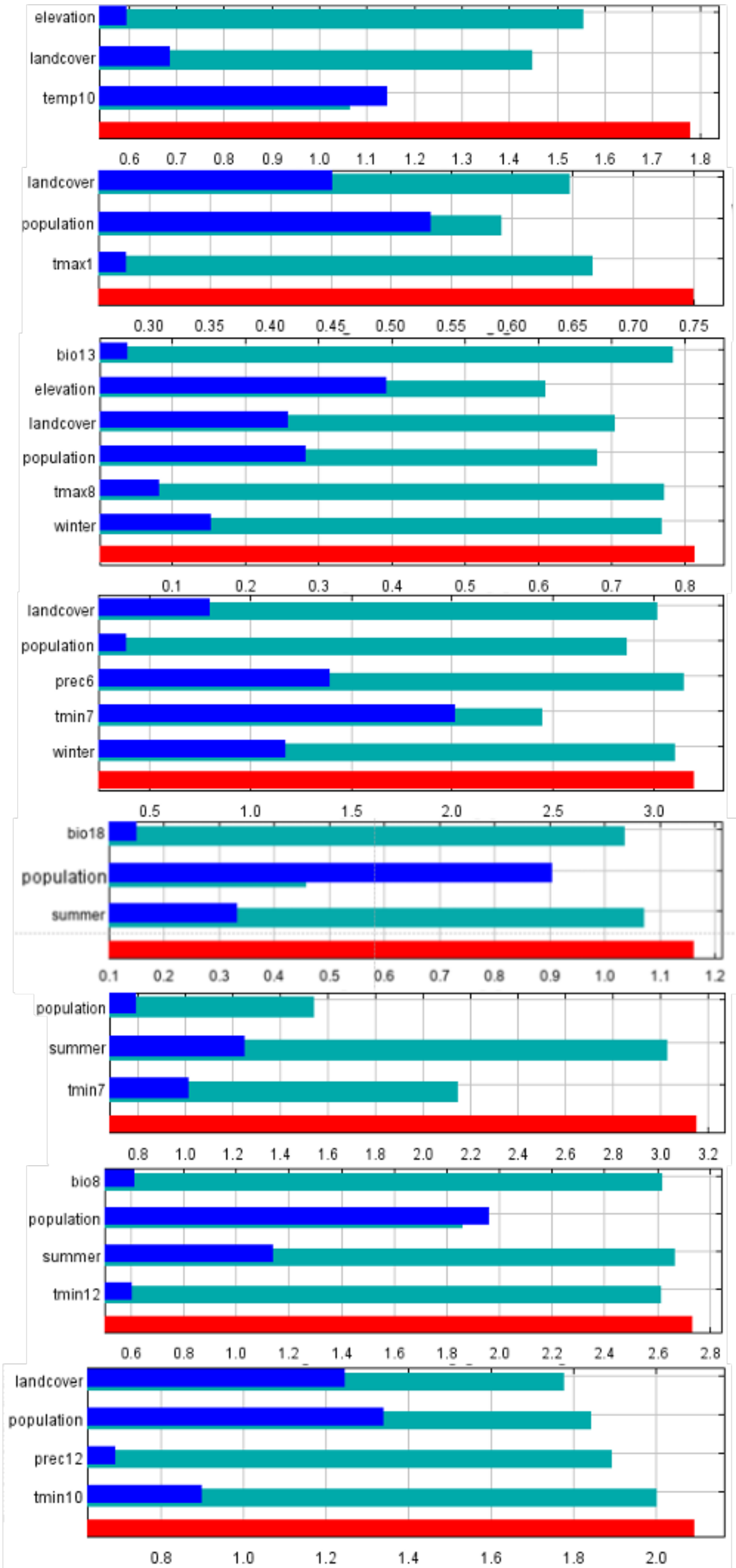

Figure S16. The Jackknife of SDM 9-16

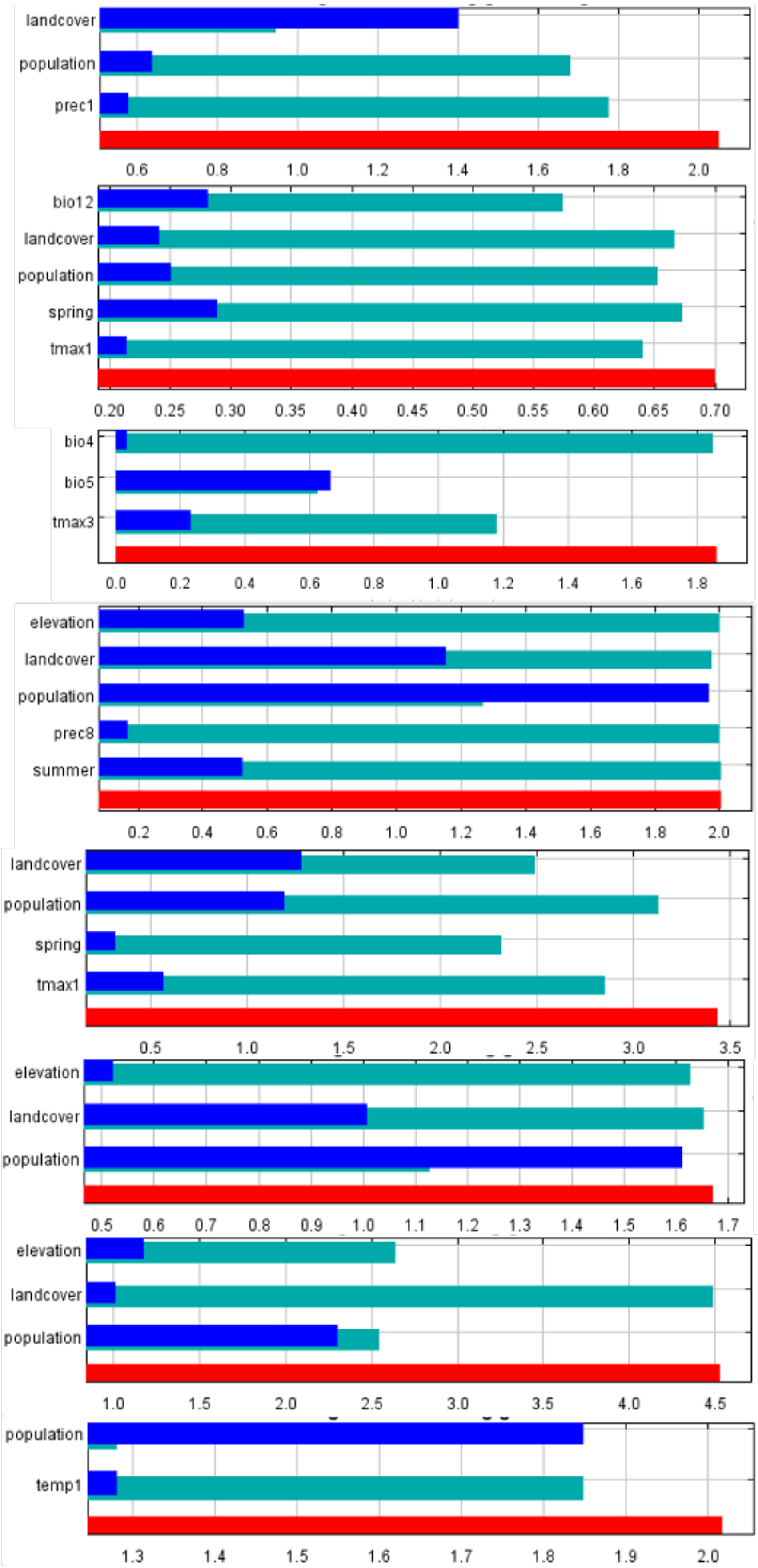

Figure S17. The Jackknife of SDM 17-24

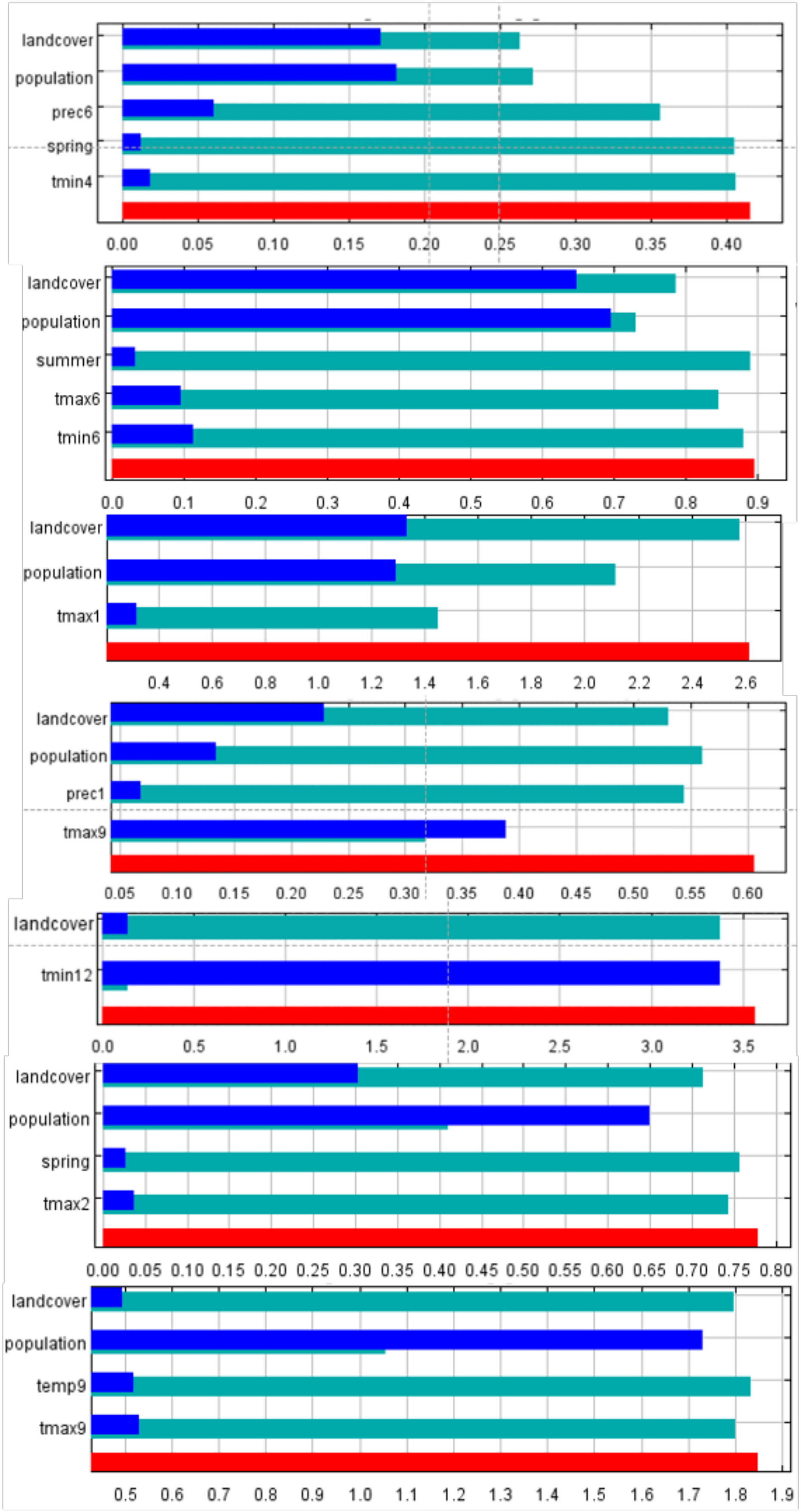

Figure S18. The Jackknife of SDM 25-31

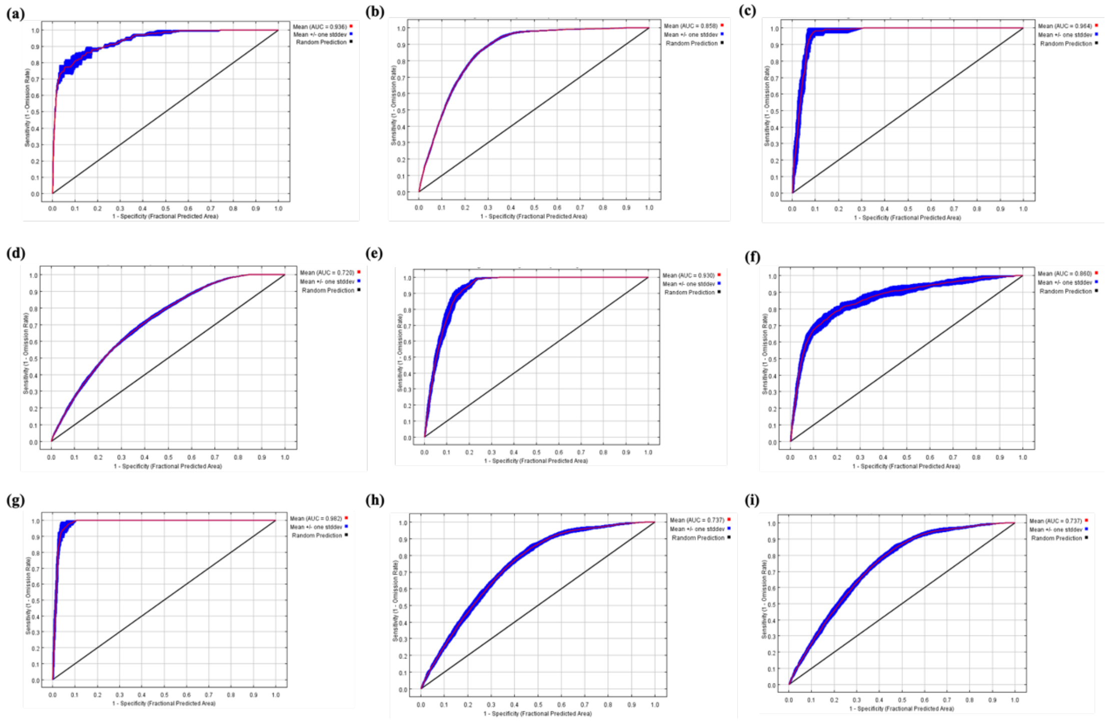

Figure S19. The ROC curves of prediction result of SDM 1-9

Sensitivity equals the proportion of test localities correctly predicted present (1–extrinsic omission rate). The quantity (1–specificity) equals the proportion of all map pixels predicted to have suitable conditions for the species.

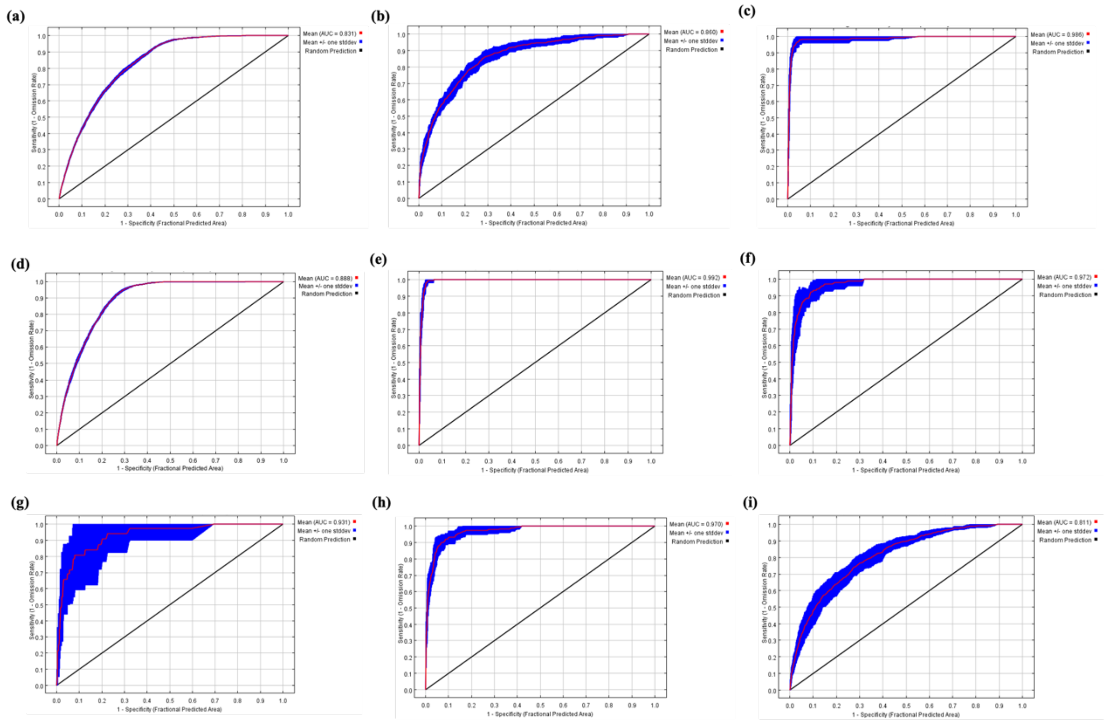

Figure S20. The ROC curves of prediction result of SDM 10-18

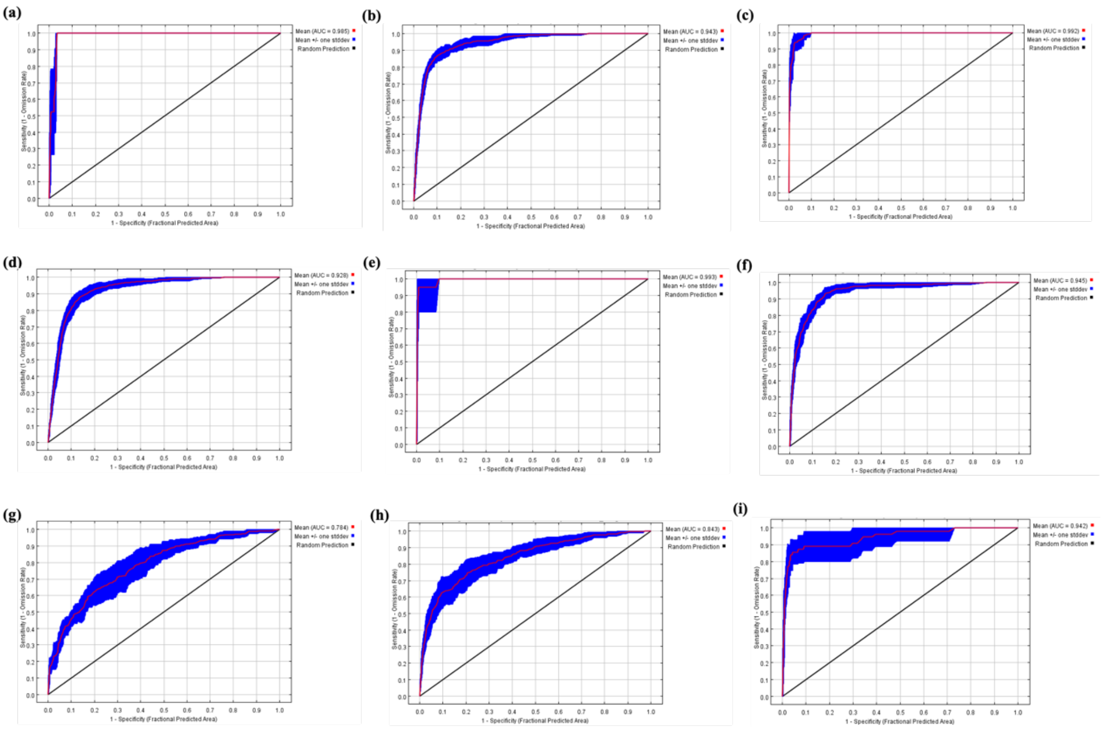

Figure S21. The ROC curves of prediction result of SDM 19-27

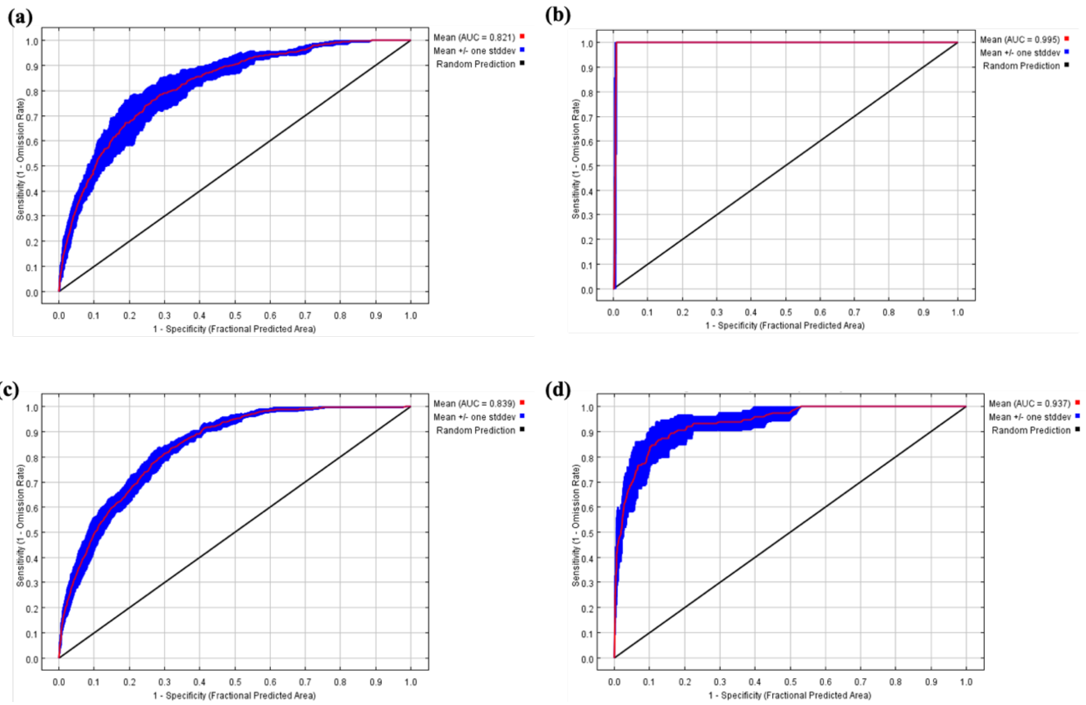

Figure S22. The ROC curves of prediction result of SDM 28-31

Table S1 The meaning of 19 bioclimatic variables used in the MaxEnt models

Bio1	Annual Mean Temperature
Bio2	Mean Diurnal Range (Mean of monthly (max temp - min temp))
Bio3	Isothermality (BIO2/BIO7)
Bio4	Temperature Seasonality
Bio5	Max Temperature of Warmest Month
Bio6	Min Temperature of Coldest Month
Bio7	Temperature Annual Range (BIO5-BIO6)
Bio8	Mean Temperature of Wettest Quarter
Bio9	Mean Temperature of Driest Quarter
Bio10	Mean Temperature of Warmest Quarter
Bio11	Mean Temperature of Coldest Quarter
Bio12	Annual Precipitation
Bio13	Precipitation of Wettest Month
Bio14	Precipitation of Driest Month
Bio15	Precipitation Seasonality (Coefficient of Variation)
Bio16	Precipitation of Wettest Quarter
Bio17	Precipitation of Driest Quarter
Bio18	Precipitation of Warmest Quarter
Bio19	Precipitation of Coldest Quarter
